# New 24-Membered Macrolactines from an Arctic Bacterium *Bacillus amyloliquefaciens* SCSIO 41392 and Their Anti-Pathogenicity Evaluation

**DOI:** 10.3390/md22110484

**Published:** 2024-10-28

**Authors:** Yue Song, Yachun Zhou, Mengjing Cong, Shengyi Deng, Yushi Chen, Xiaoyan Pang, Yonghong Liu, Li Liao, Liang Yang, Junfeng Wang

**Affiliations:** 1 CAS Key Laboratory of Tropical Marine Bio-Resources, Ecology/Guangdong Key Laboratory of Marine Materia Medica, South China Sea Institute of Oceanology, Chinese Academy of Sciences, Guangzhou 510301, China; songyue202205@163.com (Y.S.); c3021632921@163.com (M.C.); dengsy114514@outlook.com (S.D.); cyyyyys@outlook.com (Y.C.); xypang@scsio.ac.cn (X.P.); yonghongliu@scsio.ac.cn (Y.L.); 2 University of Chinese Academy of Sciences, 19 Yuquan Road, Beijing 100049, China; 3 Joint Laboratory of Guangdong-Hong Kong Universities for Vascular Homeostasis and Diseases, Department of Pharmacology, School of Medicine, Southern University of Science and Technology, Shenzhen 518055, China; zhouyc@szu.edu.cn; 4 Key Laboratory for Polar Science, Ministry of Natural Resources, Polar Research Institute of China, Shanghai 200136, China; 5 Key Laboratory of Polar Ecosystem and Climate Change, Ministry of Education, Shanghai Key Laboratory of Polar Life and Environment Sciences, School of Oceanography, Shanghai Jiao Tong University, Shanghai 200030, China

**Keywords:** macrolactines, *Bacillus amyloliquefaciens*, arctic bacteria, bacterial virulence inhibition activity

## Abstract

Three new 24-membered macrolactines, amylomacrolactines A–C (**1**–**3**), along with two known compounds **4** and **5**, were isolated from the Arctic bacteria *Bacillus amyloliquefaciens* SCSIO 41392. The configurations of **1**–**3** were assigned by a combination of coupling constants, NOESY, and analysis of MM2-optimized conformation, as well as by comparison with reports in the literature. Compounds **1** and **2** showed quorum sensing (QS) inhibitory activities against the *Pseudomonas aeruginosa (P. aeruginosa)* PQS system and suppressed PQS-regulated virulence factor pyocyanin synthesis. In addition, compounds **3**–**5** affected the production of another essential virulence factor, siderophore of pyoverdine (PVD), in *P. aeruginosa*. More importantly, compound **5** showed an anti-biofilm activity against *P. aeruginosa*. Altogether, the isolated compounds displayed multiple bacterial virulence inhibition activities, which is worthy of further exploration for novel analogues in antimicrobial drug development.

## 1. Introduction

Currently, polar regions have experienced climate changes, such as global warming and an increase in the duration of ice-free periods [1]. Much effort has been focused on research concerning microorganisms in polar regions. The e-book *Microbial Communities of Polar and Alpine Soils* aimed to collect original and noteworthy research papers about the diversity and functionality of soil microbial communities and their interactions with the other biotic components, including their adaptation and resilience abilities in stressful conditions and during environmental changes [2]. Because of their extreme environment, polar marine microorganisms are considered to be an underexplored source of novel antimicrobial compounds, which meet the need of confronting new multidrug-resistant pathogens [3,4].

*Bacillus amyloliquefaciens* SCSIO 41392 is a kind of marine bacteria isolated from Arctic samples. Macrolactins are 22- to 25-membered polyketides that are usually isolated from *Bacillus* sp. These compounds have obvious antimicrobial activity because of their specific chemical structure [5]. Most macrolactins are 24-membered, including macrolactins A, F, G, and I–L [6]. Their potent antibacterial activities against *Staphylococcus aureus* and *Bacillus subtilis* have been reported [7,8]. In spite of this, bacteria develop resistance and aggressiveness through quorum sensing (QS), which is a crucial communication form between bacteria via diffused signal molecules that enables global gene regulation and orchestrates joint actions such as motility, biofilm formation, sporulation, and virulence [9,10,11]. QS is a vital tool used by *P. aeruginosa* in leading nosocomial infections, which involved multiple virulence systems, including 3 set of QS (Las, Pqs and rhl) and Gac-Rsm two-component systems (TCSs). QS has a strong ability to form complex biofilms [12,13,14]. Siderophores are specialized small molecules produced by bacteria and fungi to facilitate the acquisition of iron from various environments, which play a vital role in the virulence of pathogens [15]. Pyoverdine (PVD), a siderophore produced by *P. aeruginosa*, is associated with biofilm formation, host pathogenicity, and virulence [16,17]. Consequently, novel antimicrobial therapies, including discovering and developing natural products that target the QS system, PVD production, and biofilm formation, have been a promising approach to tackle antimicrobial resistance in this organism [18,19].

**Figure 1:**
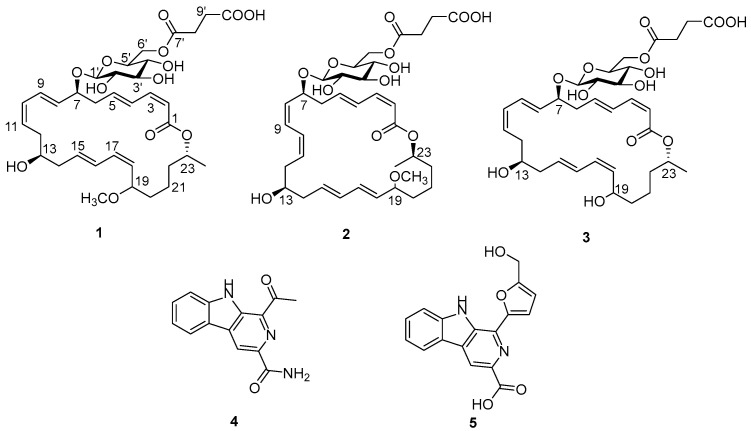
Chemical structures of compounds **1**–**5**.

During our ongoing investigations of the microorganisms inhabiting polar environments [3,20,21,22], a large scale of fermentation of a *Bacillus amyloliquefaciens* SCSIO 41392 collected from polar regions and subsequent purification has led to three new 24-membered macrolactins, amylomacrolactines A–C (**1**–**3**), and two reported compounds, stellarine A (**4**) [23] and 9 *H*-pyrido[3,4-*b*] indole-3-carboxylic acid (**5**) [24] (Figure 1). Herein, we report the isolation, structure elucidation, and the quorum-sensing inhibitory (QSI) activity of these compounds.

## 2. Results and Discussion

Compound **1** was obtained as a yellow solid. The molecular formula C_35_H_50_O_13_ was established upon analysis of the HRESIMS peak at *m/z* 677.3194 [M − H]^−^, indicating 11 degrees of unsaturation.

**Figure 2:**
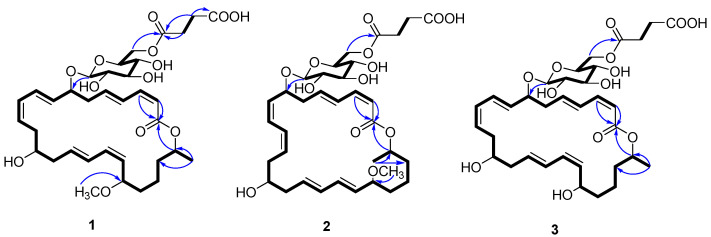
Key COSY (bold) and HMBC (arrows) correlations of **1**–**3**.

**Figure 3:**
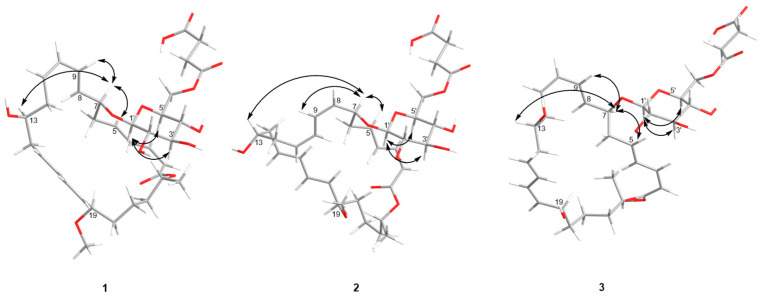
Key NOESY (double arrow) correlations of compounds **1**–**3**.

The UV absorptions at 228 and 260 nm implied the presence of an extended conjugated system. The ^1^H and ^13^C NMR spectroscopic data, including DEPT, suggested the presence of twelve sp^2^ olefinic methines, six sp^3^ methylenes, one sp^3^ methyl, four oxygenated methines, a methoxy, a lactone carbonyl carbon, and a sugar moiety. The ^13^C NMR data (Table 1) revealed two ester carbonyl carbon resonances at *δ*_C_ 167.8 and *δ*_C_ 174.5, twelve olefinic methine carbons between *δ*_C_ 117.5 and 146.0 assigned to six double bonds, nine oxygenated methine carbons between *δ*_C_ 70.6 and 101.2, one oxygenated carbon at *δ*_C_ 64.6, and nine aliphatic carbons between *δ*_C_ 21.0 and 41.1. The NMR signals (Table 1) of **1** resembled those of methoxy-macrolactin 3 [25], except for the addition of a succinic acid. In the macrolactin ring, six double bonds, one ester carbonyl carbon, and its ring accounted for a total of eight degrees of unsaturation and the remaining three degrees of unsaturation, of which two were attributed to a succinic acid, leaving one degree of unsaturation for the cyclic structure of glucose moiety, which together account for the 11 degrees of unsaturation required by the molecular formula of **1**. A cross-peak between H_2_-8′ and H_2_-9′ was observed in the COSY spectrum, while C-7′/C-8′/C-9′/C-10′ were linked by the H_2_-8′/C-7′ (*δ*_C_ 174.5), H_2_-6′ (*δ*_H_ 4.24, 4.40)/C-7′, and H_2_-9′/C-10′ (*δ*_C_ 175.2) HMBC correlation, indicating the presence of a succinic acid moiety (Figure 2). The geometric configurations of double bonds were assigned as 2*Z*,10*Z*,17*Z*, 4*E*, 8*E*, and 15*E* by the coupling constants of H-2 (*J*_2,3_ = 11.2 Hz), H-10 (*J*_10,11_ = 11.1 Hz), H-17 (*J*_17,18_ = 10.2 Hz), H-4 (*J*_4,5_ = 15.3 Hz), H-8 (*J*_8,9_ = 15.4 Hz), and H-15 (*J*_15,16_ = 15.2 Hz), respectively. Although it is controversial to assign the relative configuration of conformationally flexible macrorings based on the nuclear Overhauser effect (NOE) correlation [26], the correlations observed could provide evidence to support the relative configuration of **1**, as depicted in Figure 3. H-7*α* was assigned by correlations of H-7 with H-9 and H-5 [24,26,27]. In addition, H-13*α* was assigned by correlations of H-13 with H-7, which was in agreement with the MM2-optimized conformation of the macrocyclic nucleus [26,28] (Figure 3).

Appropriate ^1^H NMR resonances for a *β*-pyranose sugar, including the anomeric (axial) proton at *δ*_H_ 4.33, were also observed. Coupling constants analysis revealed diaxial couplings ranging from 7.8 to 7.9 Hz between all of the glycoside ring protons, thus defining the presence of *β*-glucopyranosyl moiety, which was also supported by NOESY correlations (H-1′/H-5′ and H-1′/H-3′) (Figure 3) [29,30,31,32]. The stereochemical configurations of methoxy-macrolactin 3 were confirmed as 7*S*, 13*S*, and 23*R* by analyses of ^13^C-acetonide, oxidative degradation, and chemical correlation [25,26,27]. As many macrolactin derivatives were discovered and their absolute configurations are conserved in the family, the configurations of C-7, C-13, and C-23 of **1** were suggested to be *S*, *S,* and *R* because of their similar ^1^H and ^13^C chemical shifts at the position and optical rotation values with the reported macrolactins [25,27]. Hence, the structure of **1** was identified to be a novel derivative of methoxy-macrolactin 3 with a succinic acid group at C-6′.

Compound **2** was isolated as a yellow solid with the molecular formula C_35_H_50_O_13_, as determined by a HRESIMS peak at *m/z* 677.3182 [M − H]^−^, indicating 11 degrees of unsaturation. The 1D NMR data (Table 1) of **2**, together with ^1^H − ^1^H COSY and HSQC data, were highly similar to those of methoxy-macrolactin 3 [25], except for the addition of a succinic acid, and the *J* values at H-8 (*J*_8,9_ = 11.1 Hz) and H-17 (*J*_17,18_ = 15.3 Hz) were not compatible with those of the reported macrolactin, indicating that **2** had different geometric configurations of double bonds from that of methoxy-macrolactin 3. Accordingly, the configurations of Δ^8(9)^ and Δ^17(18)^ double bonds were suggested to be *Z* and *E*, respectively. The NOESY spectrum of **2** showed cross-peaks between H-13 (*δ*_H_ 3.76) and H-7 (*δ*_H_ 4.34) that indicated H-13 and H-7 were on the same side (Figure 3) [26,27,28]. The ^13^C resonances of C-23 (*δ*_C_ 71.8) and ^1^H resonances of H-23 (*δ*_H_ 5.01, ddd (9.9, 6.4, 4.0) were similar to those of **1**, meaning **1** and **2** were likely produced by a common biosynthetic pathway [8,33,34]. Therefore, it can be assumed that the absolute configuration of C-23 in **2** was *R*. Thus, compound **2** was identified to be a novel geometric isomer of **1** with *Z* configuration at Δ^8(9)^ double bond and *E* configuration at Δ^17(18)^ double bond.

Compound **3** was obtained as a yellow solid and had a molecular formula of C_34_H_48_O_13_ as determined by HRESIMS (*m/z* 663.3039 [M − H]^−^), suggesting 11 degrees of unsaturation. Compound **3** was analogous to compound **1** except for the presence of a hydroxyl group at C-19 (*δ*_C_ 73.8) as a replacement of the methoxy group at the same position in **1** (Table 1). Moreover, the absence of the methoxy group was confirmed by the 1D NMR data (Table 1). Consequently, compound **3** was identified to be a new derivative of **1** as well.

**Figure 4:**
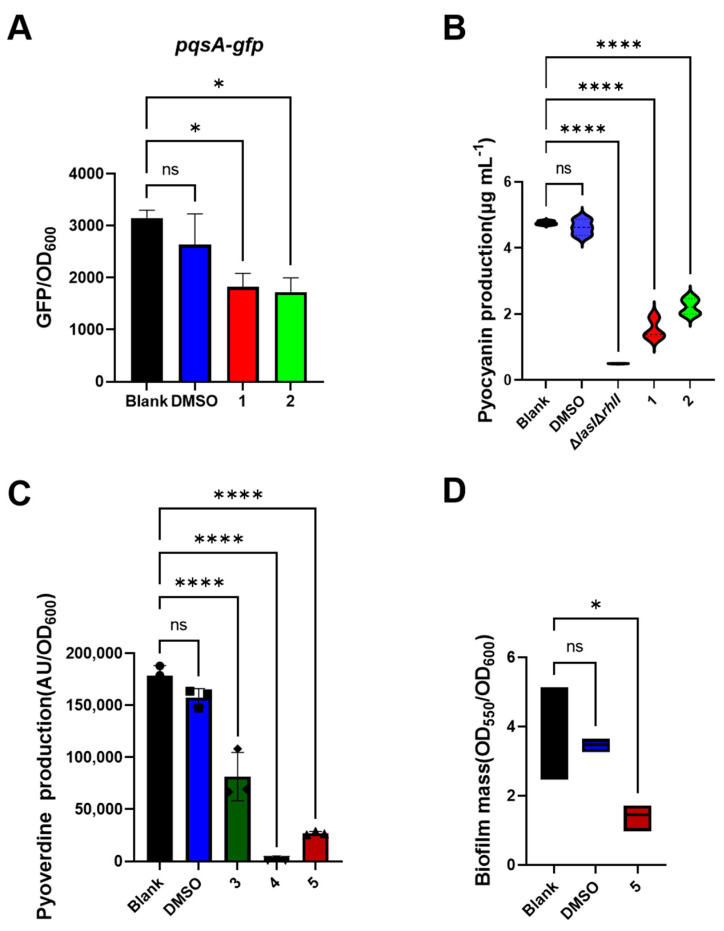
Effects of compounds **1**–**5** on various virulence phenotypes of virulence phenotype of *P. aeruginosa*. (**A**) Activity of **1** and **2** on PQS QS system. (**B**) Effects of **1** and **2** on pyocyanin production. (**C**) Impact of **3**, **4** and **5** on pyoverdine production. (**D**) Anti-biofilm activity of **5**. All compounds were dissolved in DMSO and tested at final concentration of 50 μg mL^−1^. PAO1-Δ*lasI*Δ*rhlI* was used as negative control. Error bars indicate means ± SDs. * = *p* < 0.05, **** = *p* < 0.0001, ns denotes no significance, one-way ANOVA was employed for statistic analysis.

Since macrolide antibiotics such as erythromycin and azithromycin have a Gac-Rsm-dependent inhibitory effect on various virulence phenotypes of *P. aeruginosa* [35,36], we next aimed to test the activity of compounds **1**–**5** by carried out a series of virulence assay based on the regulatory network of Gac-Rsm, including QS, PVD production, and biofilm formation [37,38]. As presented in Figure 4, both compounds **1** and **2** showed an excellent inhibition activity of the PQS QS system (Figure 4A) and PQS-related virulence factor pyocyanin of *P. aeruginosa* (Figure 4B). In addition, compounds **3**, **4,** and **5** had an active inhibitory effect on PVD production of *P. aeruginosa* (Figure 4C). Furthermore, compound **5** exhibited an efficient anti-biofilm activity (Figure 4D). Our results strongly suggested that all the compounds, especially 1 and 5, can be attractive candidates to develop resistant-robust drugs and new antimicrobial treatments due to their anti-pathogenic activity.

Although the antibacterial activities of macrolide derivatives were reported, no obvious antimicrobial activities were detected for compounds **1**–**3**. It was reported that the position of a hydroxyl group at C-15 may play an important role in the antibacterial activity of macrolactins [39]. However, none of the new compounds were hydroxylated at C-15, which may be the reason why they did not display obvious antibacterial activities.

## 3. Materials and Methods

### 3.1. General Experimental Procedures

The UV and CD spectra were recorded on a Shimadzu UV-2600 PC spectrometer (Shimadzu, Kyoto, Japan) and a Chirascan circular dichroism spectrometer (Applied Photophysics, Surrey, UK), respectively. The 1D and 2D NMR spectra were recorded on a Bruker AC 500 and 700 NMR (Bruker, Falländen, Switzerland) spectrometer with TMS as the internal standard. HRESIMS spectra were measured with a Bruker micro TOF-QII (Bruker, Fallanden, Switzerland) mass spectrometer in positive/negative ion mode. Silica gel GF-254 (10–40 mm) was used for thin-layer chromatography (TLC) (Qingdao Marine Chemical Factory, Qingdao, China). HPLC was performed using an octadecylsilyl (ODS) column (YMC-Pack ODS-A, YMC Co. Ltd. (Kyoto, Japan), 250 × 10 mm i.d., S-5 µm, 12 nm). All solvents were analytical-grade (Tianjin Fuyu Chemical and Industry Factory, Tianjin, China. The fermentation culture medium and reagents were obtained from Guangzhou Haili Aquarium Technology Company, Guangzhou, China.

### 3.2. Bacteria Strain

The bacteria strain *Bacillus amyloliquefaciens* SCSIO 41392 was isolated from deep-sea sediments over 2000 m below sea level in the Arctic Ocean (75°00.507′ N 162°01.744′ W). The isolated bacteria strain was stored on ISP Medium 2 agar (yeast extract 6 g, malt extract 10 g, glucose 12 g, agar 18 g, sea salt 30 g, water 1 L, and pH 7.2) at 28 °C and deposited in the CAS Key Laboratory of Tropical Marine Bio-resources and Ecology, South China Sea Institute of Oceanology, Chinese Academy of Sciences, Guangzhou, PR China. The 16S sequence region (1490 base pairs (bp), GenBank Accession No. KY357290.1) of strain SCSIO 41392 was amplified via the PCR process. DNA sequencing showed that it shared significant identity (100%) with *Bacillus amyloliquefaciens*.

### 3.3. Fermentation and Extraction

A few loops of cells of the strain SCSIO 41392 were inoculated into a 1 L Erlenmeyer flask containing 300 mL of seed medium (malt extract 1%, yeast extract 0.6%, glucose 1.2%, sea salt 3%, and pH 7.2) and cultivated on a rotary shaker at 180 rpm and 28 °C for 48 h as a seed culture. Then, a large-scale fermentation of the bacteria strain SCSIO 41392 was incubated in 1 L conical flasks, containing a liquid medium (300 mL/flask) composed of 4 g glucose, 4 g yeast extract, 10 g malt extract, 2 g CaCO_3_, and 1 L 3% (NaCl 3 g/H_2_O 100 mL) artificial seawater. After cultivating on a rotary shaker at 180 rpm and 28 °C for 9 days, the fermented material from each flask was extracted successively with EtOAc (700 mL/flask). Finally, the EtOAc (52.5 L) solution was concentrated under reduced pressure to obtain a dark chocolate-brown extract (43.3 g).

### 3.4. Isolation and Purification

Crude extract was separated using ODS silica gel chromatography eluted with a gradient of MeOH/H_2_O (0–100%) to yield the fractions (Fr.1–Fr.7). Fr.5 was divided into 6 parts (Fr.5.1–5.6) by using semi-preparative HPLC (34% MeCN/+0.6% FA H_2_O, 3.0 mL/min). Fr.5.3 was further purified with semi-preparative HPLC (30% MeCN/+0.6% FA H_2_O, 3.0 mL/min) to gain **5** (7.46 mg, *t*_R_ = 22 min). Fr.5.5 was further purified with semi-preparative HPLC (55% MeCN/+0.6% FA H_2_O, 2.5 mL/min) to gain **4** (1.52 mg, *t*_R_ = 13.1 min). Fr.5.4 was further separated with semi-preparative HPLC (40% MeCN/+0.6% FA H_2_O, 3.0 mL/min) to yield the fractions (Fr.5.4.1–Fr.5.4.7). With semi-preparative HPLC (42% MeCN/+0.6% FA H_2_O, 3.0 mL/min), Fr.6 was further separated to gain **2** (2.93 mg, *t*_R_ = 35.5 min) and other six fractions (Fr.6.1–Fr.6.6). Fr.6.6 was further purified with semi-preparative HPLC (35% MeCN/+0.8% FA H_2_O, 3.0 mL/min) to gain **1** (1.1 mg, *t*_R_ = 32.7 min). Compound **3** (0.86 mg, *t*_R_ = 10.5 min) was isolated from Fr.6.3 by semi-preparative HPLC (39% MeCN/+0.6% FA H_2_O, 3.0 mL/min).

Amylomacrolactine A (**1**): yellow solid; ${\left[\alpha \right]}_{D}^{25}$ − 54° (*c*, 0.1, MeOH); UV (MeOH) *λ*_max_ (log *ε*) 230 (3.18), 262 (2.81) nm; ^1^H and ^13^C NMR data, Table 1; HR-ESI-MS *m/z* 677.3194 [M − H]^−^ (calcd for C_35_H_49_O_13_^−^, 677.3179), 1355.6452 [2M − H]^−^ (calcd for C_70_H_99_O_26_^−^, 1355.6430).

Amylomacrolactine B (**2**): yellow solid; ${\left[\alpha \right]}_{D}^{25}$ − 32° (*c*, 0.1, MeOH); UV (MeOH) *λ*_max_ (log *ε*) 228 (3.12), 260 (2.78) nm; ^1^H and ^13^C NMR data, Table 1; HR-ESI-MS *m/z* 677.3182 [M − H]^−^ (calcd for C_35_H_49_O_13_^−^, 677.3179).

Amylomacrolactine C (**3**): yellow solid; ${\left[\alpha \right]}_{D}^{25}$ − 42° (*c*, 0.04, MeOH); UV (MeOH) *λ*_max_ (log *ε*) 228 (3.39), 262 (3.09) nm; ^1^H and ^13^C NMR data, Table 1; HR-ESI-MS *m/z* 663.3039 [M − H]^−^ (calcd for C_34_H_47_O_13_^−^, 663.3022), 699.2818 [M + Cl]^−^ (calcd for C_34_H_48_ClO_13_^−^, 699.2789).

### 3.5. P. aeruginosa QS Inhibition Assay

All the compounds were dissolved in DMSO with a stock concentration of 10 mg mL^−1^ unless otherwise stated. The QS inhibition assay was conducted as previously described (Table S1). Briefly, the optical density at 600 nm (OD_600_) of an overnight culture of the PAO1-*pqsA-gfp* strain (grown in LB broth medium at 37 °C, 200 rpm) was adjusted to 0.01 with ABTGC medium in the 96-well microtiter plate and the compound was added with a final concentration of 50 μg mL^−1^. Both the DMSO group and blank group were set as controls. The microtiter plate was further incubated at 37 °C in a Tecan Infinite 200 Pro plate reader (Tecan Group Ltd., Mannedorf, Switzerland) to measure OD_600_ and GFP fluorescence with excitation at 485 nm and emission at 535 nm for 20 h. All experiments were performed in triplicate.

### 3.6. Pyocyanin Quantification Assay

Both the OD_600_ of PAO1 and Δ*lasI*Δ*rhlI* mutant strains were standardized to 0.01 in the cell culture tube with a volume of 3.5 mL ABTGC medium; after the compounds was added (50 μg mL^−1^), the mixture was further cultured at 37 °C with 200 rpm for 20 h. The mutant strain Δ*lasI*Δ*rhlI* and the DMSO group were used as negative control. The cultures were then detected for cell density (OD_600_) followed by centrifugation for 10 min at 10,000 rpm. The resulting supernatant was used for pyocyanin extraction with chloroform (3 mL) and 0.2 M HCl (1.5 mL). Finally, the top aqueous layer of HCl containing pyocyanin was pipetted into a microtiter plate and measured at 520 nm. The pyocyanin value was calculated as OD_520/_OD_600_.

### 3.7. Biofilm Formation Assay

Overnight cultures of PAO1 were diluted in ABTGC medium at a cell density of OD_600_ equal to 0.01 in 96-well microtiter plates. Then, the compound (50 μg mL^−1^) or DMSO was added, and the plate was incubated at 37 °C without agitation for 24 hours. OD_600_ of suspended cultures was measured by Tecan Infinite 200 Pro plate reader (Tecan Group Ltd., Mannedorf, Switzerland), after which we removed the liquid cultures and washed the plate with phosphate-buffered saline (PBS) to remove remaining suspended cells. Then, the biofilms were stained with 1.0% crystal violet for 15 min followed by washing twice with PBS. The crystal violet-stained biofilms were eluted by 100% ethanol and the absorbance of biofilm-associated dye was measured at 550 nm. The formation of biofilms was normalized by the amount of suspended cells (OD at 600 nm).

### 3.8. Pyoverdine Production Assay

Pyoverdine level was determined according to a reported method with modify [40]. Overnight cultures of PAO1 were suspended in fresh ABTGC medium with compounds or DMSO at a cell density of OD_600_ = 0.01 in 96-well microtiter plates. Then, the plate was put into a Tecan Infinite 200 Pro plate reader (Tecan Group Ltd., Mannedorf, Switzerland) to record OD_600_ and the pyoverdine level with a parameter of excitation at 398 nm and emission at 460 nm for 22 h. The production of pyoverdine was calculated by dividing pyoverdine data by OD_600_.

### 3.9. Statistical Analysis

Statistical analysis was conducted by GraphPad Prism (GraphPad Prism 8.1.2; GraphPad Software, San Diego, CA, USA). One-way ANOVA analysis was used to evaluate the significance within groups. Statistical significance was determined at *p* < 0.05. All experiments were performed in triplicate at minimum, and the results are shown as the mean ± sd.

## 4. Conclusions

In conclusion, three new 24-membered macrolactones, macrolactins **1**–**3**, were isolated from the Arctic bacteria *Bacillus amyloliquefaciens* SCSIO 41392. Compounds **1**–**5** were tested for various virulence phenotypes of *P. aeruginosa*, and the data showed that compounds **1** and **2** exhibited PQS QS inhibitory activity, compounds **3**–**5** efficiently inhibited the production of PVD, and compound **5** effectively impaired the formation of biofilm. Together, our results showed that these compounds can serve as promising lead molecules in antimicrobial drug development.

## Figures and Tables

**Table 1:** ^1^H and ^13^C NMR Data of **1**–**3** (700, 175 MHz, CD_3_OD).

No.	1		2		3	
	*δ*_C_, Type	*δ*_H_ (*J* in Hz)	*δ*_C_, Type	*δ*_H_ (*J* in Hz)	*δ*_C_, Type	*δ*_H_ (*J* in Hz)
1	167.8, C		168.0, C		167.8, C	
2	117.5, CH	5.56, d (11.2)	117.7, CH	5.53, d (11.4)	117.5, CH	5.56, d (11.3)
3	146.0, CH	6.63, t (11.2)	145.5, CH	6.59, dd (15.4, 11.4)	145.9, CH	6.62, t (11.3)
4	130.1, CH	7.34, dd (15.3, 11.3)	130.4, CH	7.29, t (15.4)	130.2, CH	7.31–7.37, m
5	142.1, CH	6.15–6.18, m	141.7, CH	6.08–6.12, dd (15.4, 4.8)	142.0, CH	6.16, s
6	41.1, CH_2_	2.37, m	40.6, CH_2_	2.46, m	41.0, CH_2_	2.38, m
		2.58–2.67, m		2.62, m		2.59, m
7	80.3, CH	4.30, d (7.7)	79.8, CH	4.34, t (6.8)	80.2, CH	4.29, d (7.9)
8	133.8, CH	5.62, dd (15.4, 8.3)	133.6, CH	5.61, dd (11.1, 7.7)	133.8, CH	5.67, dd (15.3, 7.7)
9	130.1, CH	6.52, dd (15.4, 11.1)	130.0, CH	6.55, dd (11.1, 4.2)	130.2, CH	6.51, dd (15.3, 4.3)
10	130.7, CH	6.13, d (11.1)	130.6, CH	6.14, d (11.1)	130.7, CH	6.13, d (10.6)
11	129.7, CH	5.62, m	129.6, CH	5.59, m	129.6, CH	5.64, m
12	34.7, CH_2_	2.45, m	34.8, CH_2_	2.37, m	34.6, CH_2_	2.43, m
		2.33, m				2.33, m
13	72.3, CH	3.69, dd (7.1, 5.3)	71.8, CH	3.76, d (5.7)	72.3, CH	3.69, m
14	40.5, CH_2_	2.26, tq (14.3, 7.6)	40.1, CH_2_	2.26, dd (14.3, 7.5)	40.3, CH_2_	2.24, m
15	131.8, CH	5.70, dt (15.2, 7.6)	131.5, CH	5.72, dt (15.2, 7.5)	131.1, CH	5.69, m
16	133.4, CH	6.08, dd (15.2, 10.3)	133.4, CH	6.05, dd (15.2, 10.5)	133.7, CH	6.04, t (13.0)
17	135.1, CH	6.19, d (10.2)	134.7, CH	6.17, d (15.3)	132.2, CH	6.13, d (10.8)
18	132.7, CH	5.31, q (8.5)	132.5, CH	5.35, dd (15.3, 7.9)	135.5, CH	5.50, m
19	83.9, CH	3.51, dd (15.0, 6.6)	83.3, CH	3.59, dd (13.2, 7.6)	73.8, CH	3.97, d (6.9)
20	35.5, CH_2_	1.53, dtt (13.4, 10.1, 5.7)	35.8, CH_2_	1.47, dt (13.7, 7.3)	36.9, CH_2_	1.55, m
21	22.3, CH_2_	1.36, m	22.0, CH_2_	1.34–1.38, q (7.7)	22.5, CH_2_	1.39, m
22	36.9, CH_2_	1.63, m	36.8, CH_2_	1.62, m	37.3, CH_2_	1.66, m
23	70.6, CH	5.03, ddd (9.6, 6.4, 3.3)	71.8, CH	5.01, ddd (9.9, 6.4, 4.0)	70.7, CH	5.05, d (6.3)
24	21.0, CH_3_	1.22, d (6.3)	20.8, CH_3_	1.24, d (6.3)	21.0, CH_3_	1.23, m
1′	101.2, CH	4.31, d (7.8)	101.5, CH	4.33, d (7.8)	101.2, CH	4.31, d (7.8)
2′	75.0, CH	3.23, d (7.9)	75.0, CH	3.23, d (7.9)	75.0 CH	3.24, d (8.1)
3′	77.9, CH	3.35–3.37, m	77.9, CH	3.33, d (5.1)	77.9, CH	3.32, m
4′	71.5, CH	3.32, m	71.6, CH	3.32, d (5.1)	71.5, CH	3.34, m
5′	75.2, CH	3.35–3.37, m	75.3, CH	3.38, t (6.9)	75.3, CH	3.36, m
6′	64.6, CH_2_	4.24, dd (11.7, 4.3)	64.8, CH_2_	4.24, dd (11.8, 5.6)	64.6, CH_2_	4.24, d (10.0)
		4.40, d (11.7)		4.39, dd (11.8, 2.1)		4.40, d (11.6)
7′	174.5, C		174.3, C		174.6, C	
8′	30.8, CH_2_	2.62, m	30.5, CH_2_	2.62, m	30.7, CH_2_	2.62, m
9′	31.2, CH_2_	2.56, m	30.7, CH_2_	2.58, m	30.7, CH_2_	2.62, m
10′	175.2, C		176.7, C		176.8, C	
19-OCH_3_	56.3	3.21, s	56.3	3.21, s		

## Data Availability

Data is contained within the article or Supplementary Materials.

## Supplementary Materials

The following supporting information can be downloaded at: https://www.mdpi.com/article/10.3390/md22110484/s1, Table S1: The tested *P. aeruginosa* strains used in this study; Figure S1: ^1^H NMR spectrum of compound **1** (CD_3_OD, 700MHz); Figure S2: ^13^C NMR spectrum of compound **1** (CD_3_OD, 175 MHz); Figure S3: ^13^C-DEPT135 NMR spectrum of compound **1** (CD_3_OD, 175 MHz); Figure S4: HSQC spectrum of compound **1** (CD_3_OD); Figure S5: ^1^H-^1^H COSY spectrum of compound **1** (CD_3_OD); Figure S6: HMBC spectrum of compound **1** (CD_3_OD); Figure S7: NOESY spectrum of compound **1** (CD_3_OD); Figure S8: HR-ESI-MS spectrum of compound **1**; Figure S9: UV spectrum of compound **1**; Figure S10: CD spectrum of compound **1**; Figure S11: ^1^H NMR spectrum of compound **2** (CD_3_OD, 700MHz); Figure S12: ^13^C NMR spectrum of compound **2** (CD_3_OD, 175 MHz); Figure S13: ^13^C-DEPT135 NMR spectrum of compound **2** (CD_3_OD, 175 MHz); Figure S14: HSQC spectrum of compound **2** (CD_3_OD); Figure S15: ^1^H-^1^H COSY spectrum of compound **2** (CD_3_OD); Figure S16: HMBC spectrum of compound **2** (CD_3_OD); Figure S17: NOESY spectrum of compound **2** (CD_3_OD); Figure S18: HR-ESI-MS spectrum of compound **2**; Figure S19: UV spectrum of compound **2**; Figure S20: CD spectrum of compound **2**; Figure S21: ^1^H NMR spectrum of compound **3** (CD_3_OD, 700MHz); Figure S22: ^13^C NMR spectrum of compound **3** (CD_3_OD, 175 MHz); Figure S23: ^13^C-DEPT135 NMR spectrum of compound **3** (CD_3_OD, 175 MHz); Figure S24: HSQC spectrum of compound **3** (CD_3_OD); Figure S25: ^1^H-^1^H COSY spectrum of compound **3** (CD_3_OD); Figure S26: HMBC spectrum of compound **3** (CD_3_OD); Figure S27: NOESY spectrum of compound **3** (CD_3_OD); Figure S28: HR-ESI-MS spectrum of compound **3**; Figure S29: UV spectrum of compound **3**; Figure S30: CD spectrum of compound **3**.
