# Supplementary material for: New 24-Membered Macrolactines from an Arctic Bacterium Bacillus amyloliquefaciens SCSIO 41392 and Their Anti-Pathogenicity Evaluation
Source: Mar Drugs. 2024 Oct 28;22(11):484. doi: 10.3390/md22110484 (PMC11595553; doi:10.3390/md22110484)
Supplement: Supplementary file 1 [file marinedrugs-22-00484-s001.zip › marinedrugs-3264206-supplementary.pdf]

Supplementary Information

New 24-membered macrolactines from an Arctic bacteria *Bacillus amyloliquefaciens* SCSIO 41392 and their anti-pathogenicity evaluation

Yue Song[#1,4], Yachun Zhou[#2], Mengjing Cong[1,4], Shengyi Deng[1], Yushi Chen[1], Xiaoyan Pang[1], Yonghong Liu[1,4], Li Liao[*3,5], Liang Yang[*2], Junfeng Wang[*1,4]

[1] CAS Key Laboratory of Tropical Marine Bio-resources and Ecology/Guangdong Key Laboratory of Marine Materia Medica, South China Sea Institute of Oceanology, Chinese Academy of Sciences, Guangzhou 510301, China;

[2] Joint Laboratory of Guangdong-Hong Kong Universities for Vascular Homeostasis and Diseases, Department of Pharmacology, School of Medicine, Southern University of Science and Technology, Shenzhen, 518055, P. R. China;

[3] Key Laboratory for Polar Science, MNR, Polar Research Institute of China, Shanghai 200136, China;

[4] University of Chinese Academy of Sciences, 19 Yuquan Road, Beijing 100049, China;

[5] Key Laboratory of Polar Ecosystem and Climate Change, Ministry of Education, Shanghai Key Laboratory of Polar Life and Environment Sciences, School of Oceanography, Shanghai Jiao Tong University, Shanghai, 200030, China

* Correspondence: liaoli@pric.org.cn (Li Liao); yangl@sustech.edu.cn (Liang Yang); wangjunfeng@scsio.ac.cn (Junfeng Wang)

List of Supporting Information

The 16S gene sequence data of *Bacillus amyloliquefaciens* SCSIO 41392

Figure S1. ^{1}H NMR spectrum of compound **1** (CD_3OD, 700MHz).

Figure S2. ^{13}C NMR spectrum of compound **1** (CD_3OD, 175MHz).

Figure S3. ^{13}C-DEPT135 NMR spectrum of compound **1** (CD_3OD, 175MHz).

Figure S4. HSQC spectrum of compound **1** (CD_3OD).

Figure S5. ^{1}H-^{1}H COSY spectrum of compound **1** (CD_3OD).

Figure S6. HMBC spectrum of compound **1** (CD_3OD).

Figure S7. NOESY spectrum of compound **1** (CD_3OD).

Figure S8. HR-ESI-MS spectrum of compound **1**.

Figure S9. UV spectrum of compound **1**.

Figure S10. CD spectrum of compound **1**.

Figure S11. ^{1}H NMR spectrum of compound **2** (CD_3OD, 700MHz).

Figure S12. ^{13}C NMR spectrum of compound **2** (CD_3OD, 175MHz).

Figure S13. ^{13}C-DEPT135 NMR spectrum of compound **2** (CD_3OD, 175MHz).

Figure S14. HSQC spectrum of compound **2** (CD_3OD).

Figure S15. ^{1}H-^{1}H COSY spectrum of compound **2** (CD_3OD).

Figure S16. HMBC spectrum of compound **2** (CD_3OD).

Figure S17. NOESY spectrum of compound **2** (CD_3OD).

Figure S18. HR-ESI-MS spectrum of compound **2**.

Figure S19. UV spectrum of compound **2**.

Figure S20. CD spectrum of compound **2**.

Figure S21. ^{1}H NMR spectrum of compound **3** (CD_3OD, 700MHz).

Figure S22. ^{13}C NMR spectrum of compound **3** (CD_3OD, 175MHz).

Figure S23. ^{13}C-DEPT135 NMR spectrum of compound **3** (CD_3OD, 175MHz).

Figure S24. HSQC spectrum of compound **3** (CD_3OD).

Figure S25. ^{1}H-^{1}H COSY spectrum of compound **3** (CD_3OD).

Figure S26. HMBC spectrum of compound **3** (CD_3OD).

Figure S27. NOESY spectrum of compound **3** (CD_3OD).

Figure S28. HR-ESI-MS spectrum of compound **3**.

Figure S29. UV spectrum of compound **3**.

Figure S30. CD spectrum of compound **3**.

Table S1. The tested *P. aeruginosa* strains used in this study.

Physicochemical data of known compounds **4**–**5**.

The 16S gene sequence data of *Bacillus amyloliquefaciens* SCSIO 41392

CTGTTACGACTTCACCCCAATCATCTGTCCCACCTTCGGCGGCTGGCTCCAT

AAAGGTTACCTCACCGACTTCGGGTGTTACAAACTCTCGTGGTGTGACGGG

CGGTGTGTACAAGGCCCGGGAACGTATTCACCGCGGCATGCTGATCCGCGA

TTACTAGCGATTCCAGCTTCACGCAGTCGAGTTGCAGACTGCGATCCGAACT

GAGAACAGATTTGTGGGATTGGCTTAACCTCGCGGTTTCGCTGCCCTTTGTT

CTGTCCATTGTAGCACGTGTGTAGCCCAGGTCATAAGGGGCATGATGATTT

GACGTCATCCCCACCTTCCTCCGGTTTGTCACCGGCAGTCACCTTAGAGTGC

CCAACTGAATGCTGGCAACTAAGATCAAGGGTTGCGCTCGTTGCGGGACTT

AACCCAACATCTCACGACACGAGCTGACGACAACCATGCACCACCTGTCAC

TCTGCCCCCGAAGGGGACGTCCTATCTCTAGGATTGTCAGAGGATGTCAAG

ACCTGGTAAGGTTCTTCGCGTTGCTTCGAATTAAACCACATGCTCCACCGCT

TGTGCGGGCCCCCGTCAATTCCTTTGAGTTTCAGTCTTGCGACCGTACTCCC

CAGGCGGAGTGCTTAATGCGTTAGCTGCAGCACTAAGGGGCGGAAACCCCC

TAACACTTAGCACTCATCGTTTACGGCGTGGACTACCAGGGTATCTAATCCT

GTTCGCTCCCCACGCTTTCGCTCCTCAGCGTCAGTTACAGACCAGAGAGTCG

CCTTCGCCACTGGTGTTCCTCCACATCTCTACGCATTTCACCGCTACACGTG

GAATTCCACTCTCCTCTTCTGCACTCAAGTTCCCCAGTTTCCAATGACCCTCC

CCGGTTGAGCCGGGGGCTTTCACATCAGACTTAAGAAACCGCCTGCGAGCC

CTTTACGCCCAATAATTCCGGACAACGCTTGCCACCTACGTATTACCGCGGC

TGCTGGCACGTAGTTAGCCGTGGCTTTCTGGTTAGGTACCGTCAAGGTGCCG

CCCTATTTGAACGGCACTTGTTCTTCCCTAACAACAGAGCTTTACGATCCGA

AAACCTTCATCACTCACGCGGCGTTGCTCCGTCAGACTTTCGTCCATTGCGG

AAGATTCCCTACTGCTGCCTCCCGTAGGAGTCTGGGCCGTGTCTCAGTCCCA

GTGTGGCCGATCACCCTCTCAGGTCGGCTACGCATCGTCGCCTTGGTGAGCC

GTTACCTCACCAACTAGCTAATGCGCCGCGGGTCCATCTGTAAGTGGTAGCC

GAAGCCACCTTTTATGTCTGAACCATGCGGTTCAGACAACCATCCGGTATTA

GCCCCGGTTTCCCGGAGTTATCCCAGTCTTACAGGCAGGTTACCCACGTGTT

ACTCACCCGTCCGCCGCTAACATCAGGGAGCAAGCTCCCATCTGTCCGCTCG

ACTTGCATGTATTAGGCACGCCGCCAGCGTTCGTCCTGAGCA

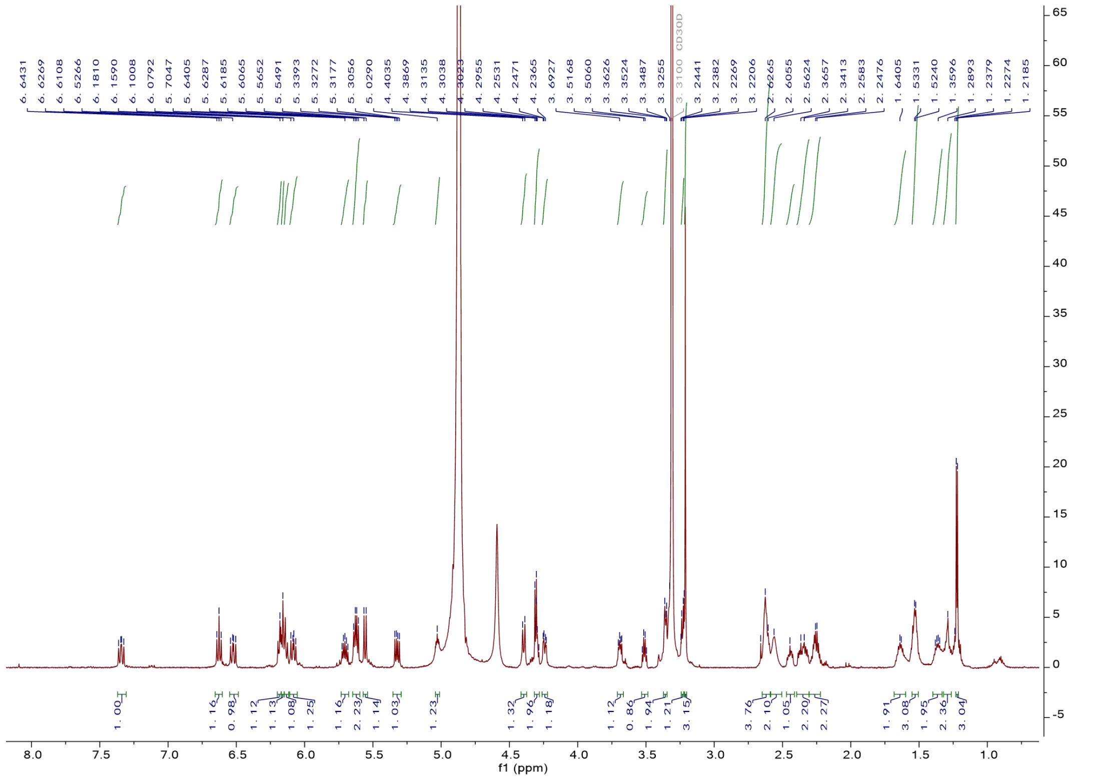

Figure S1. ^{1}H NMR spectrum of compound **1** (CD_3OD, 700MHz).

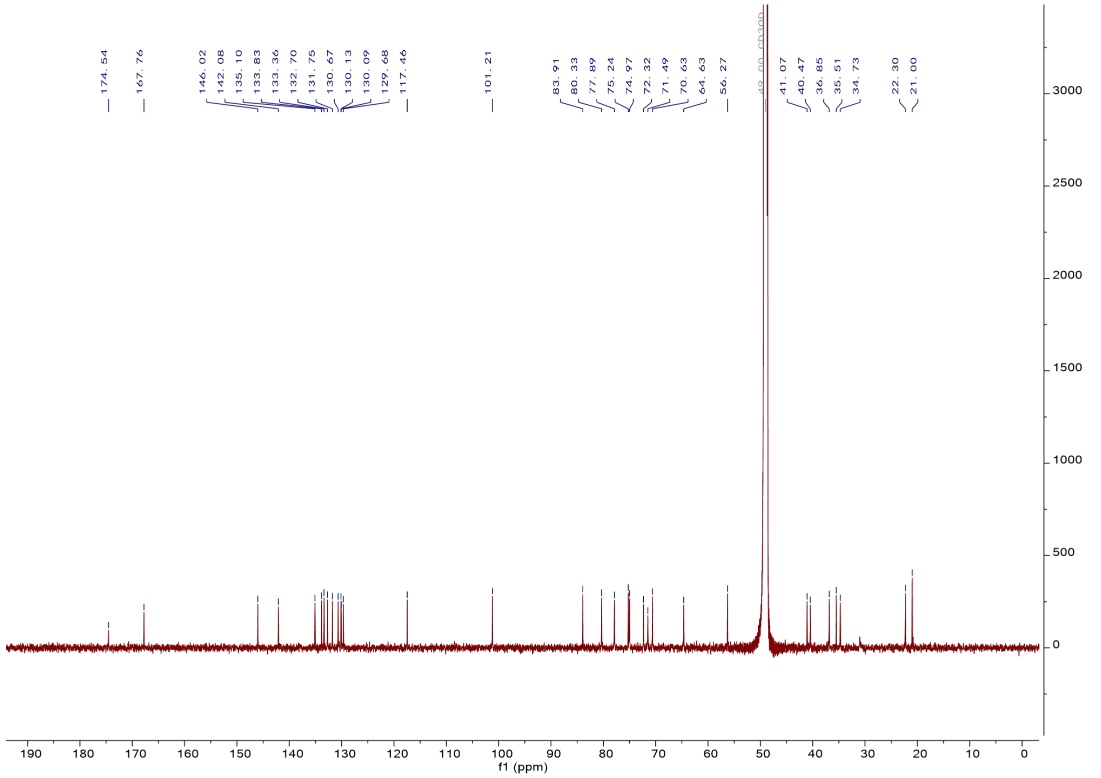

Figure S2. ^{13}C NMR spectrum of compound **1** (CD_3OD, 175MHz).

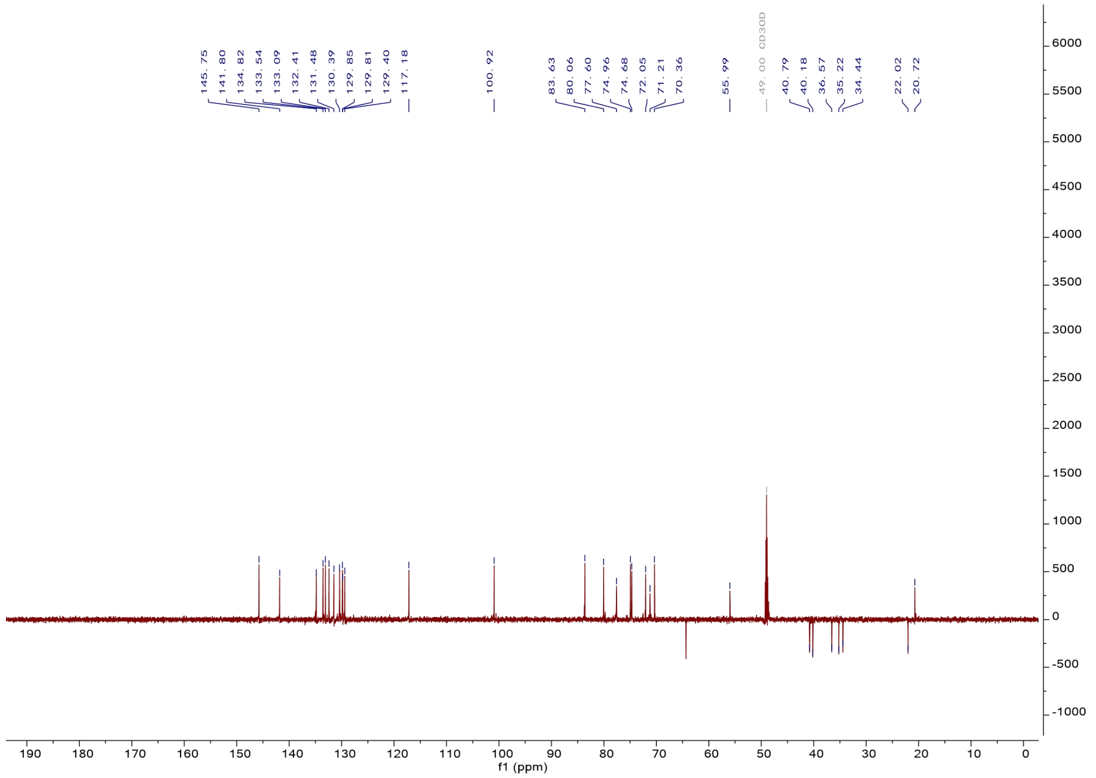

Figure S3. ^{13}C-DEPT135 NMR spectrum of compound **1** (CD_3OD, 175MHz).

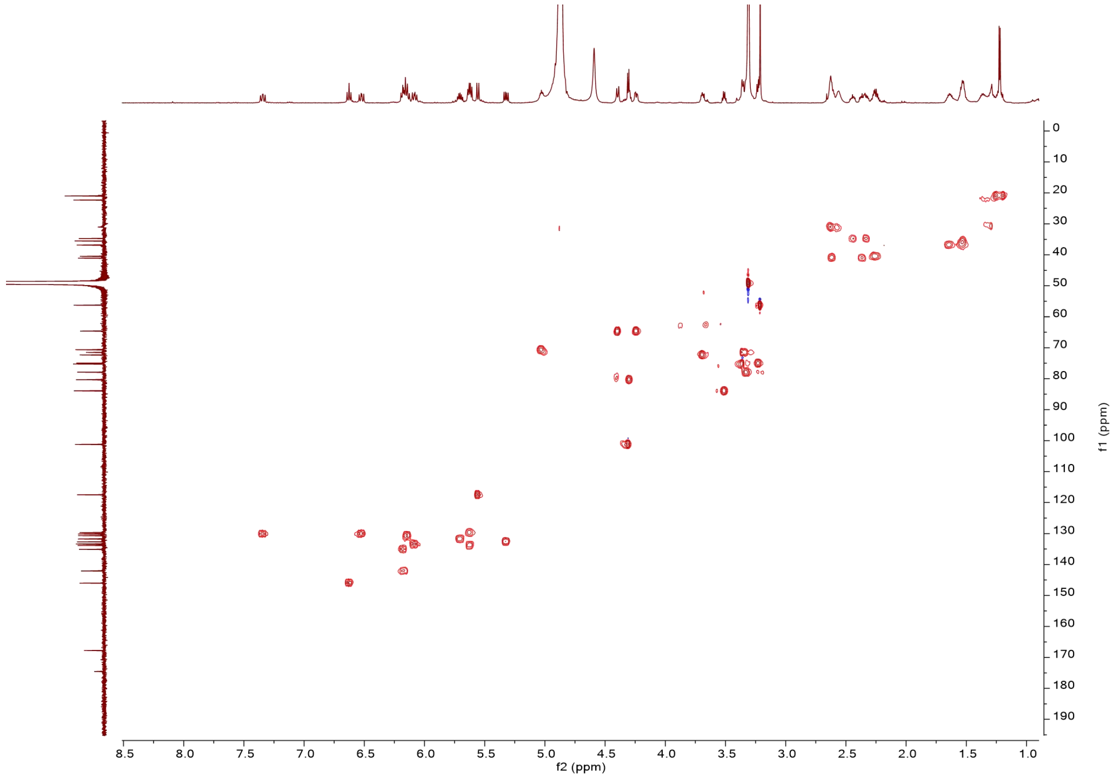

Figure S4. HSQC spectrum of compound **1** (CD_3OD).

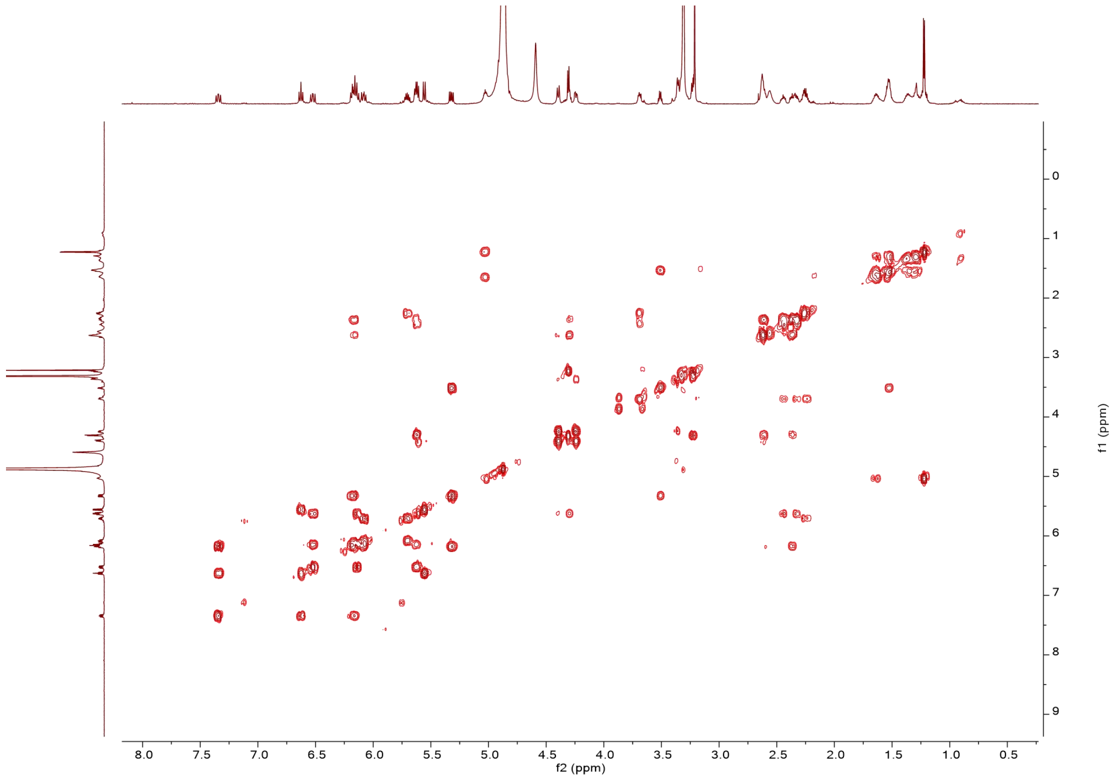

Figure S5. ^{1}H-^{1}H COSY spectrum of compound **1** (CD_3OD).

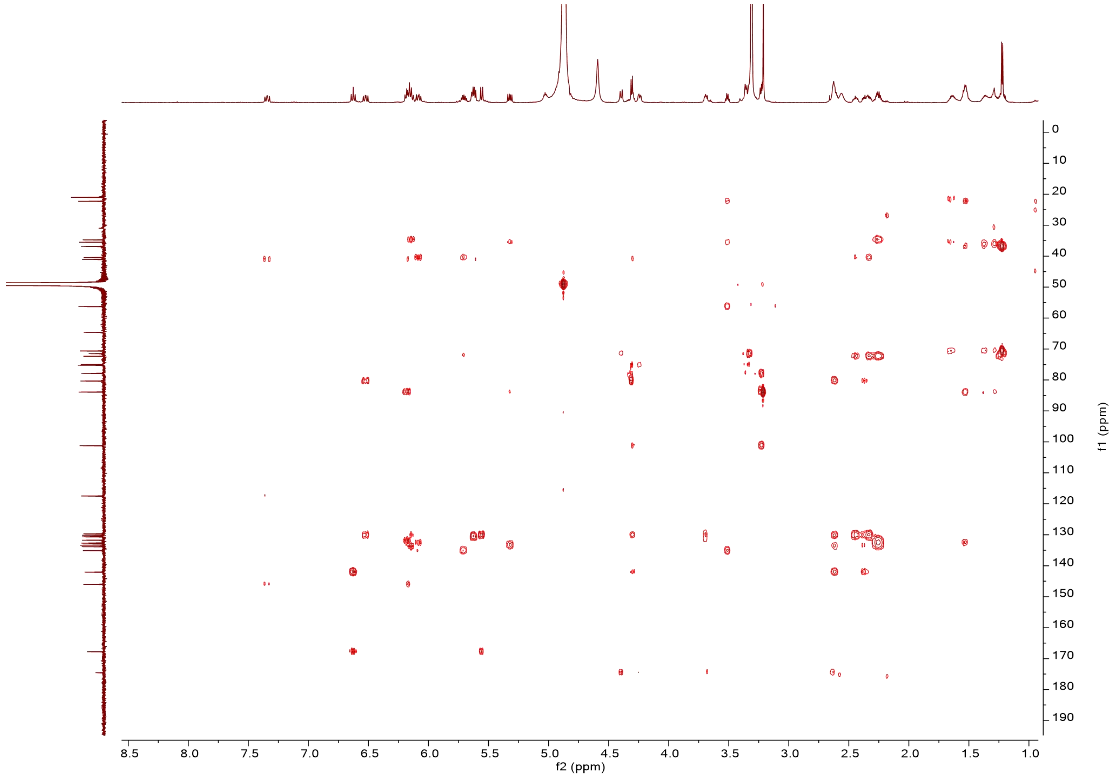

Figure S6. HMBC spectrum of compound **1** (CD_3OD).

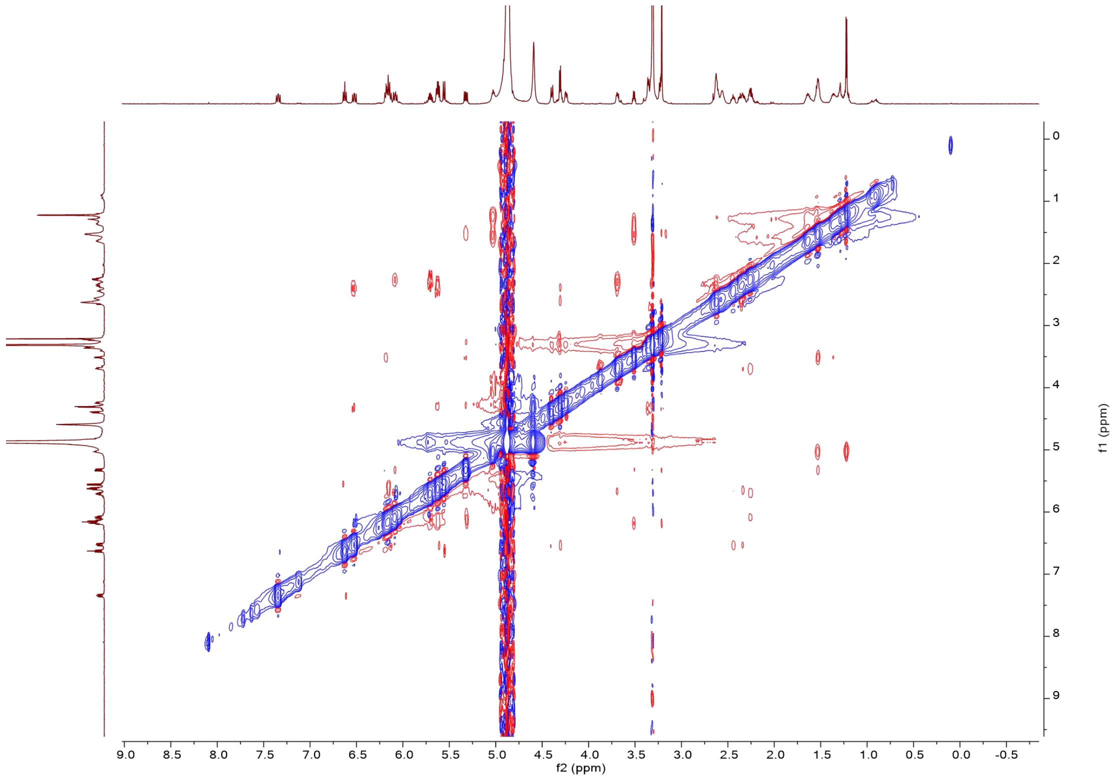

Figure S7. NOESY spectrum of compound **1** (CD_3OD).

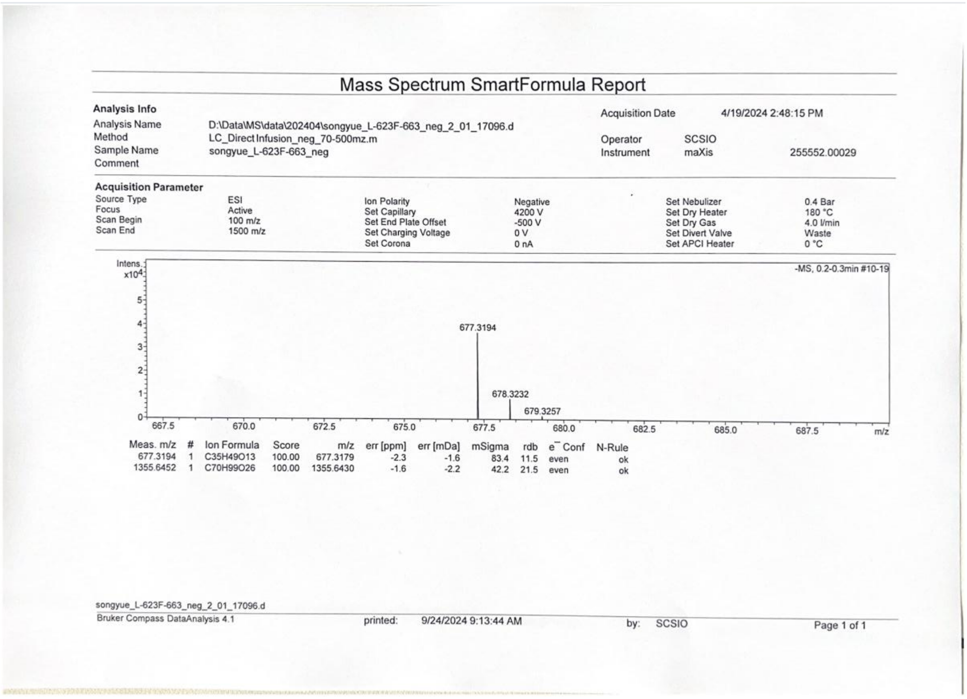

Mass Spectrum SmartFormula Report

Analysis Info

Analysis Name	D:\Data\MS\data\202404\songyue_L-623F-663_neg_2_01_17096.d	Acquisition Date	4/19/2024 2:48:15 PM	
Method	LC_Direct Infusion_neg_70-500mz.m	Operator	SCSIO	
Sample Name	songyue_L-623F-663_neg	Instrument	maXis	255552.00029
Comment				

Acquisition Parameter

Source Type	ESI	Ion Polarity	Negative	Set Nebulizer	0.4 Bar
Focus	Active	Set Capillary	4200 V	Set Dry Heater	180 °C
Scan Begin	100 m/z	Set End Plate Offset	-500 V	Set Dry Gas	4.0 l/min
Scan End	1500 m/z	Set Charging Voltage	0 V	Set Divert Valve	Waste
		Set Corona	0 nA	Set APCI Heater	0 °C

Meas. m/z	#	Ion Formula	Score	m/z	err [ppm]	err [mDa]	mSigma	rdb	e⁻ Conf	N-Rule
677.3194	1	C35H49O13	100.00	677.3179	-2.3	-1.6	83.4	11.5	even	ok
1355.6452	1	C70H99O26	100.00	1355.6430	-1.6	-2.2	42.2	21.5	even	ok

songyue_L-623F-663_neg_2_01_17096.d
Bruker Compass DataAnalysis 4.1 printed: 9/24/2024 9:13:44 AM by: SCSIO Page 1 of 1

Figure S8. HR-ESI-MS spectrum of compound **1**.

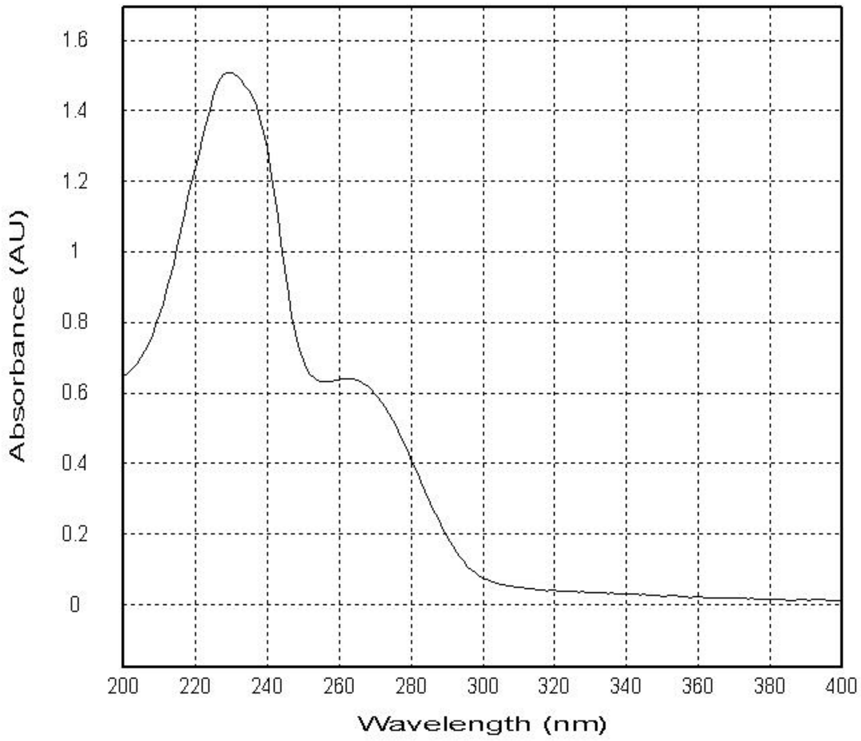

Figure S9. UV spectrum of compound **1**.

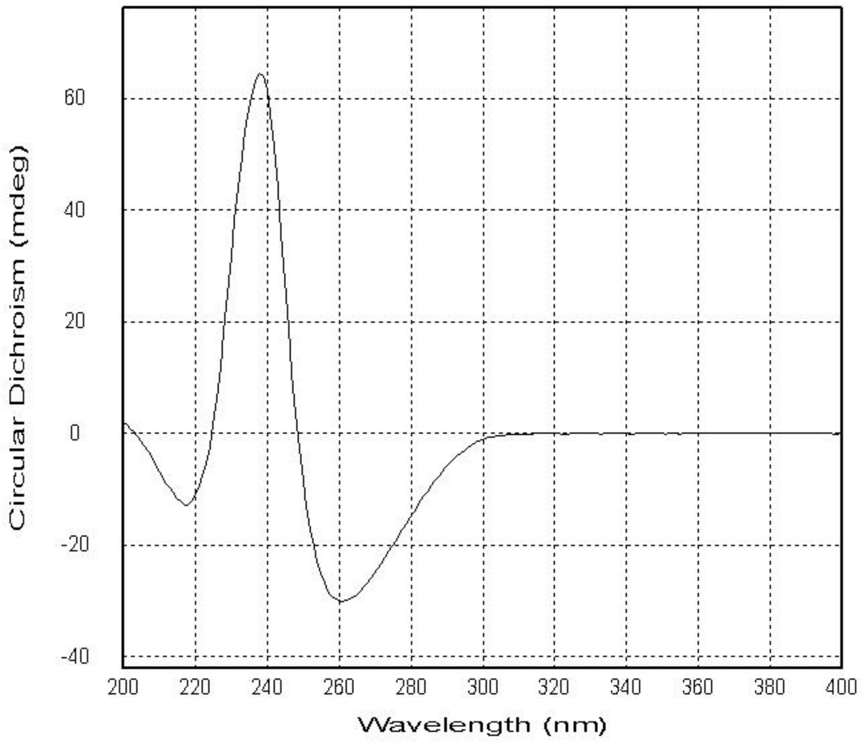

Figure S10. CD spectrum of compound **1**.

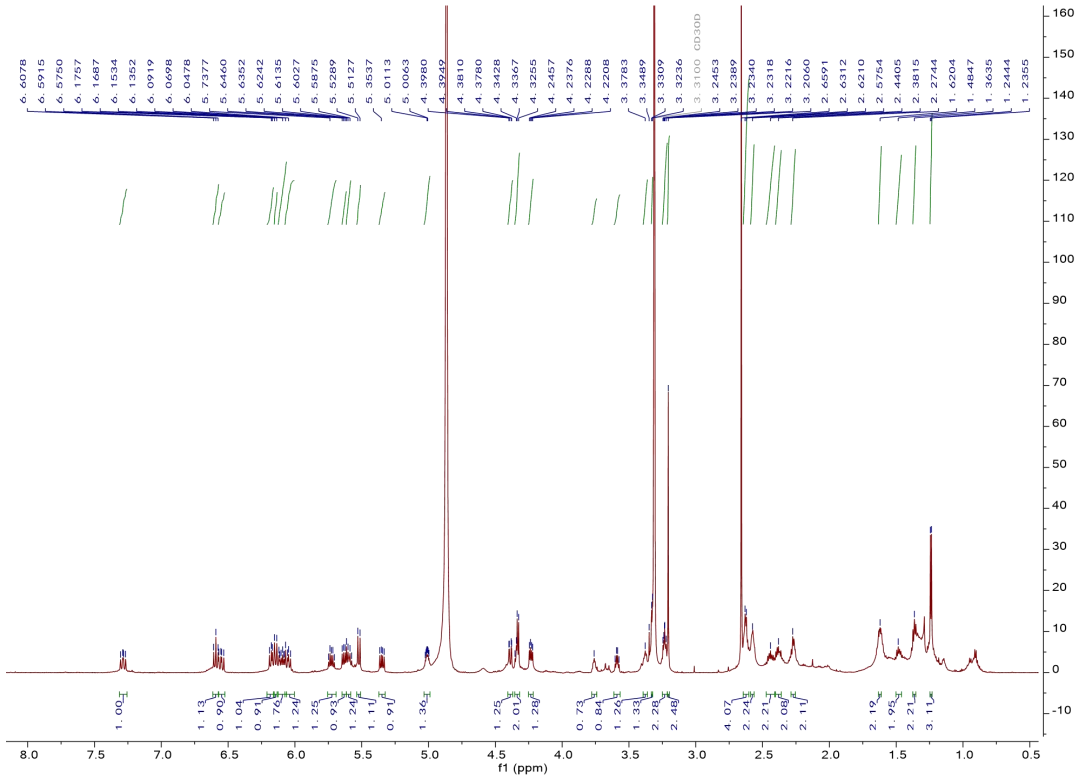

Figure S11. ^{1}H NMR spectrum of compound **2** (CD_3OD, 700MHz).

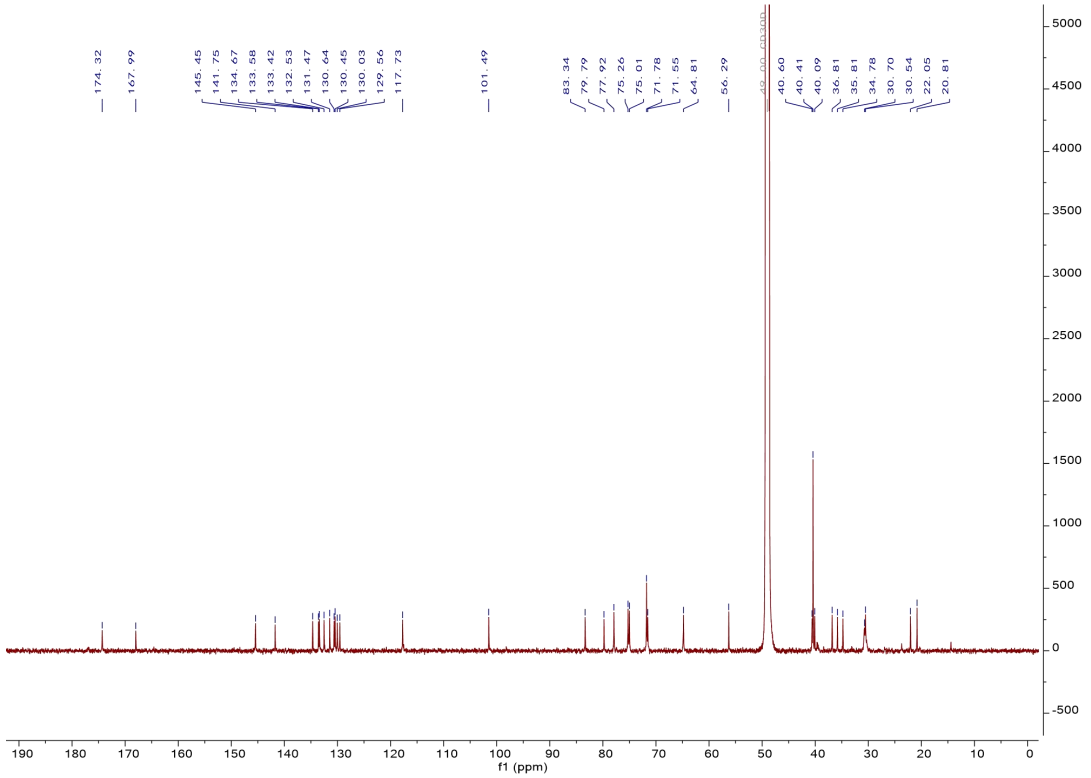

Figure S12. ^{13}C NMR spectrum of compound **2** (CD_3OD, 175MHz).

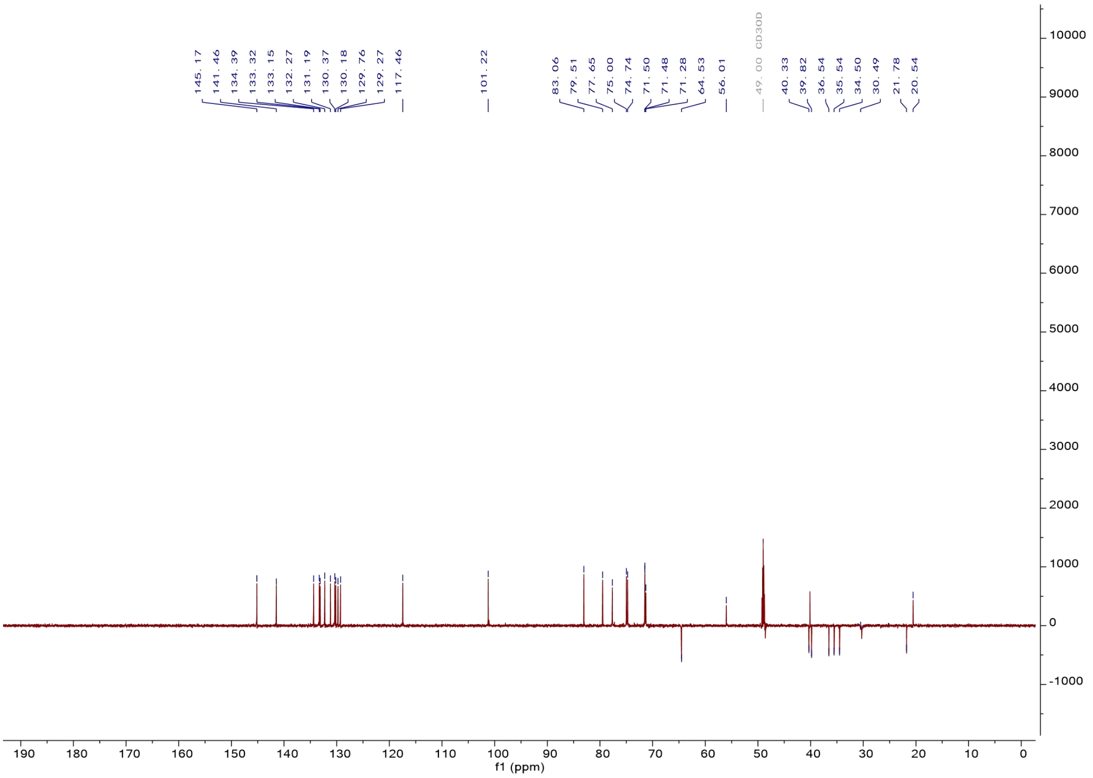

Figure S13. ^{13}C-DEPT135 NMR spectrum of compound **2** (CD_3OD, 175MHz).

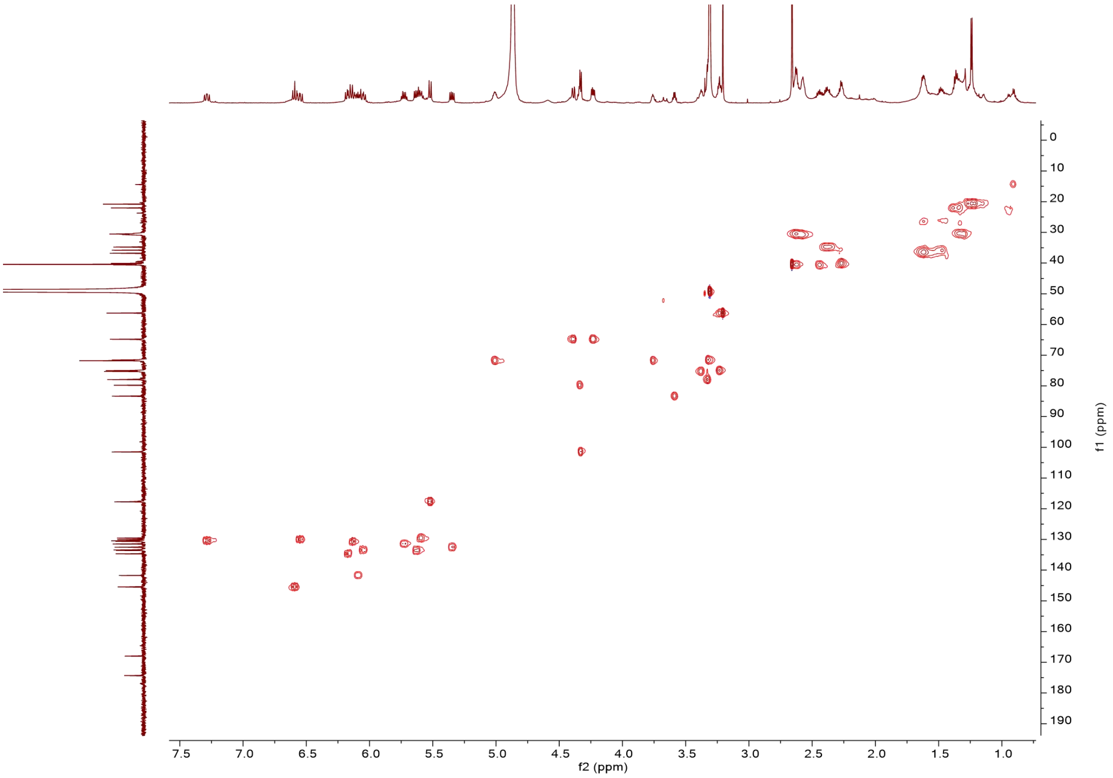

Figure S14. HSQC spectrum of compound **2** (CD_3OD).

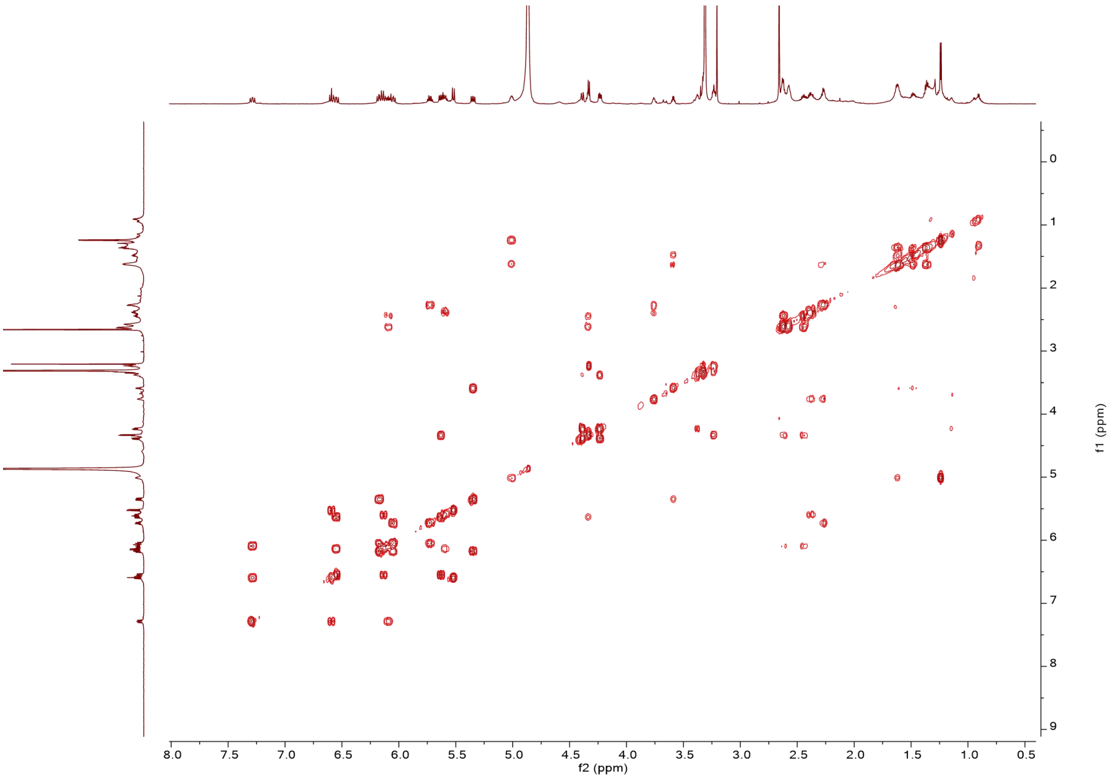

Figure S15. ^{1}H-^{1}H COSY spectrum of compound **2** (CD_3OD).

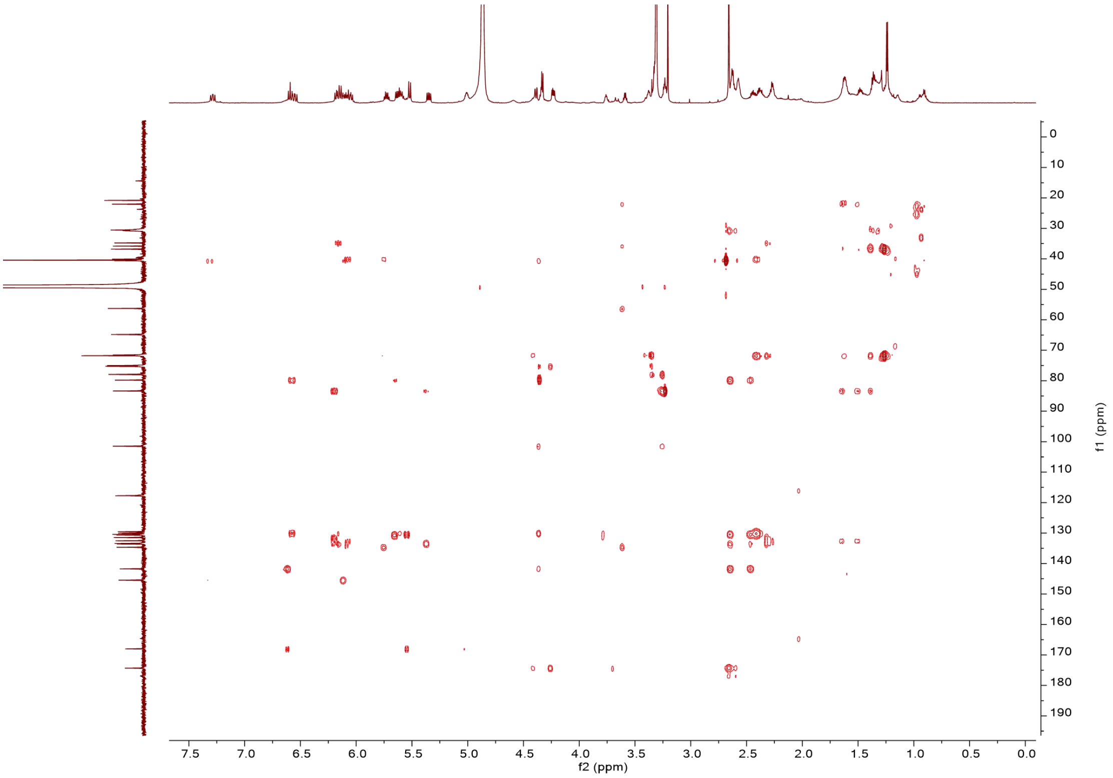

Figure S16. HMBC spectrum of compound **2** (CD_3OD).

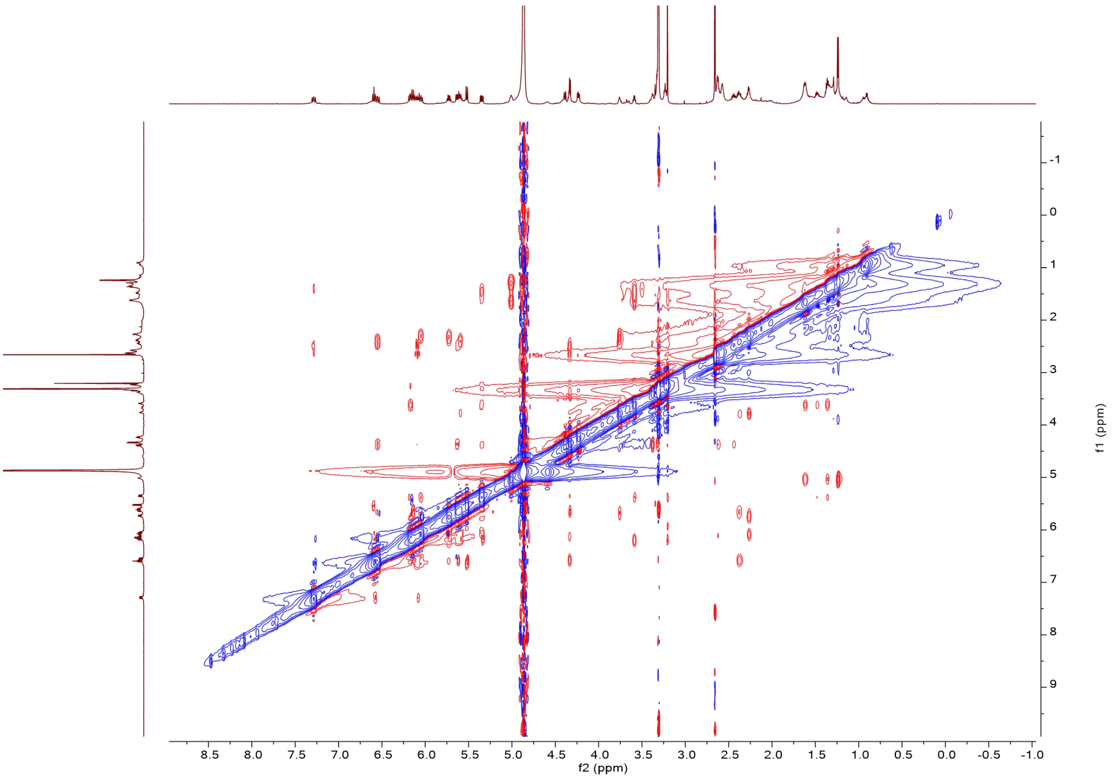

Figure S17. NOESY spectrum of compound **2** (CD_3OD).

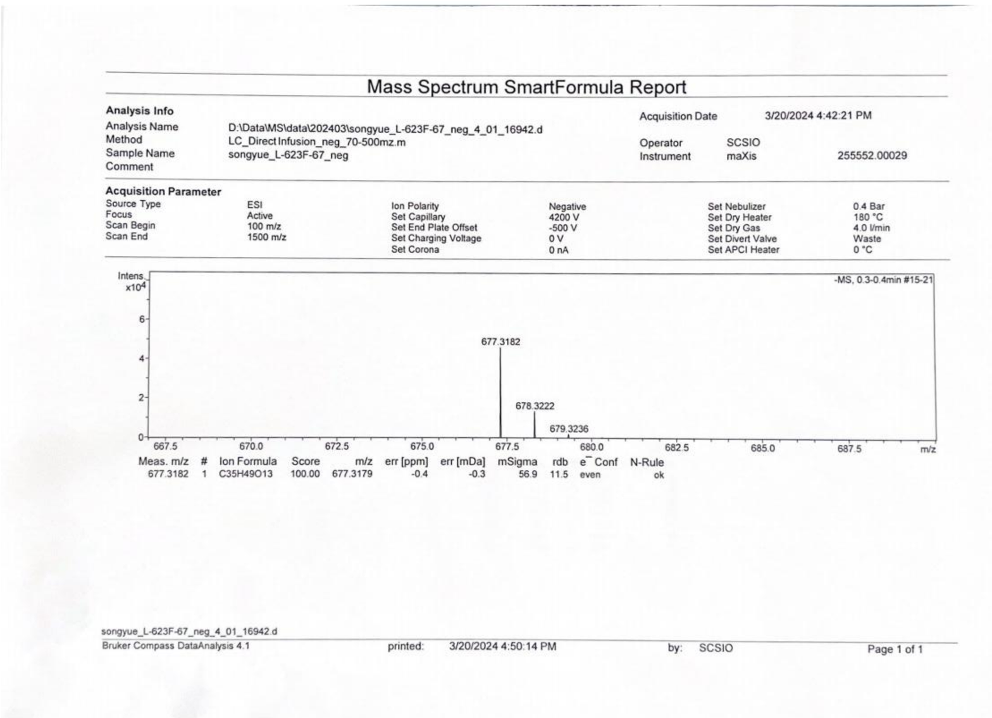

Mass Spectrum SmartFormula Report

Analysis Info			
Analysis Name	D:\Data\MS\data\202403\songyue_L-623F-67_neg_4_01_16942.d	Acquisition Date	3/20/2024 4:42:21 PM
Method	LC_Direct Infusion_neg_70-500mz.m	Operator	SCSIO
Sample Name	songyue_L-623F-67_neg	Instrument	maXis 255552.00029
Comment			

Acquisition Parameter					
Source Type	ESI	Ion Polarity	Negative	Set Nebulizer	0.4 Bar
Focus	Active	Set Capillary	4200 V	Set Dry Heater	180 °C
Scan Begin	100 m/z	Set End Plate Offset	-500 V	Set Dry Gas	4.0 l/min
Scan End	1500 m/z	Set Charging Voltage	0 V	Set Divert Valve	Waste
		Set Corona	0 nA	Set APCI Heater	0 °C

Meas. m/z	#	Ion Formula	Score	m/z	err [ppm]	err [mDa]	mSigma	rdb	e⁻ Conf	N-Rule
677.3182	1	C35H49O13	100.00	677.3179	-0.4	-0.3	56.9	11.5	even	ok

songyue_L-623F-67_neg_4_01_16942.d
Bruker Compass DataAnalysis 4.1 printed: 3/20/2024 4:50:14 PM by: SCSIO Page 1 of 1

Figure S18. HR-ESI-MS spectrum of compound **2**.

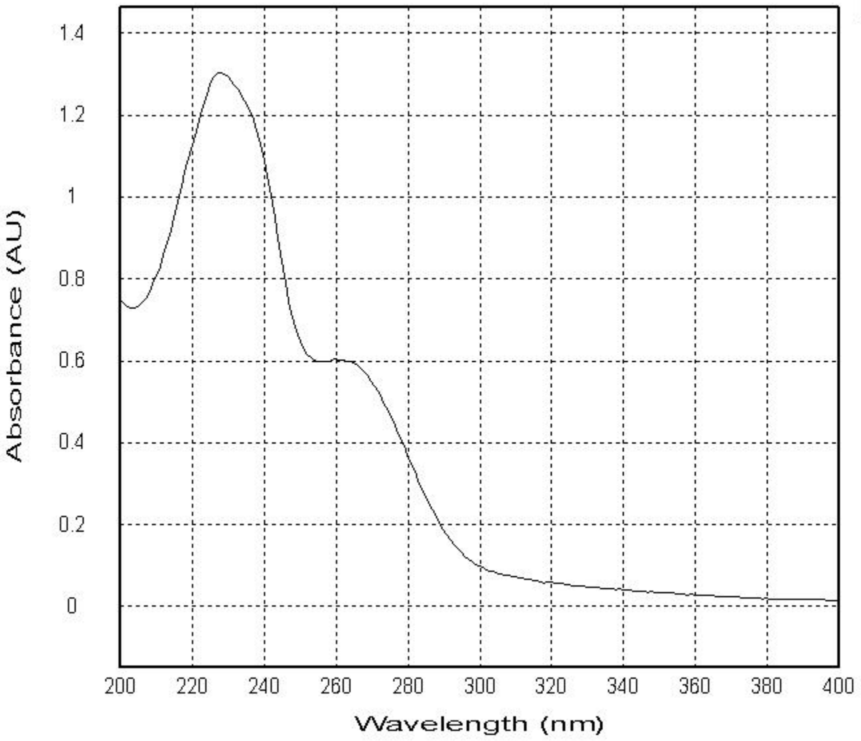

Figure S19. UV spectrum of compound **2**.

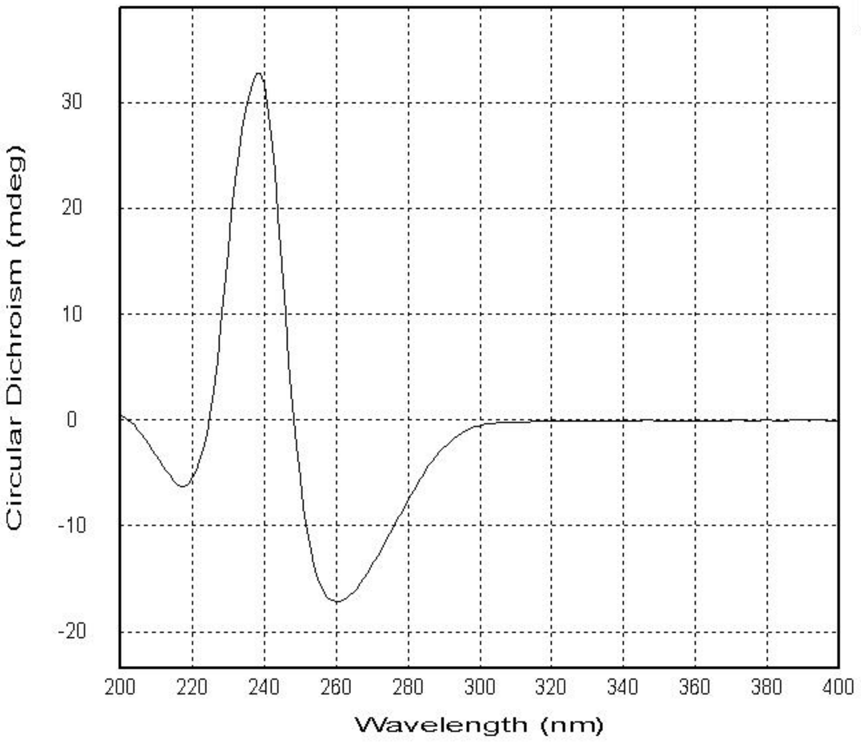

Figure S20. CD spectrum of compound **2**.

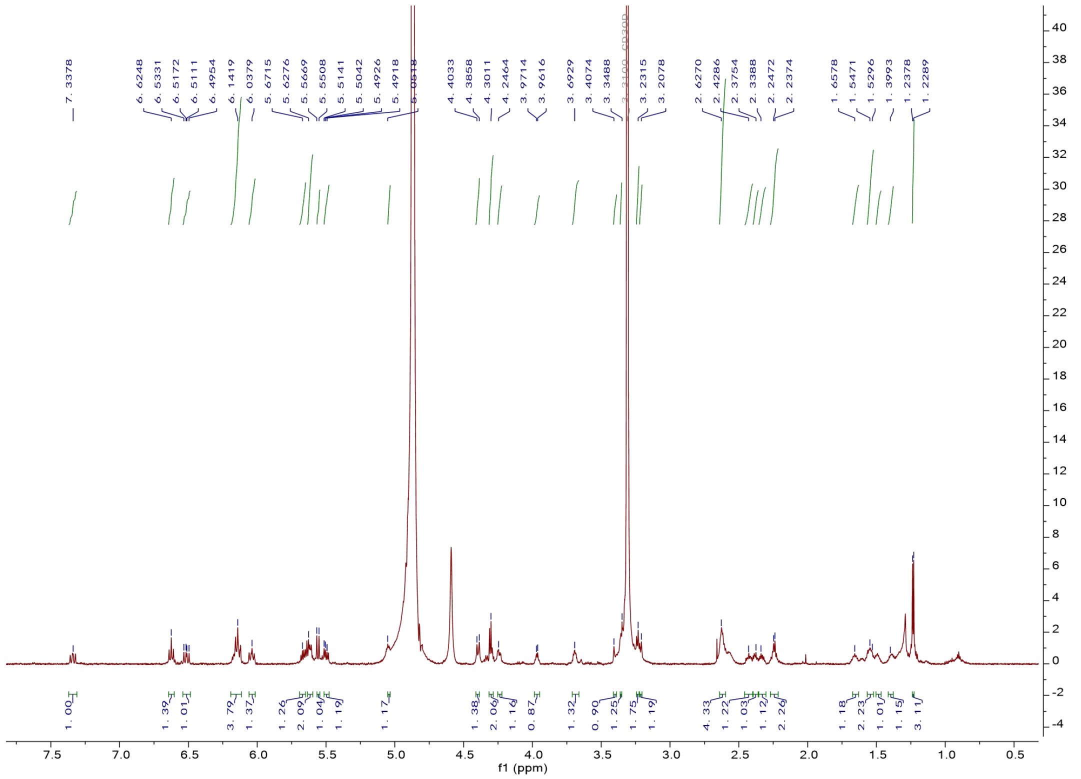

Figure S21. ^{1}H NMR spectrum of compound **3** (CD_3OD, 700MHz).

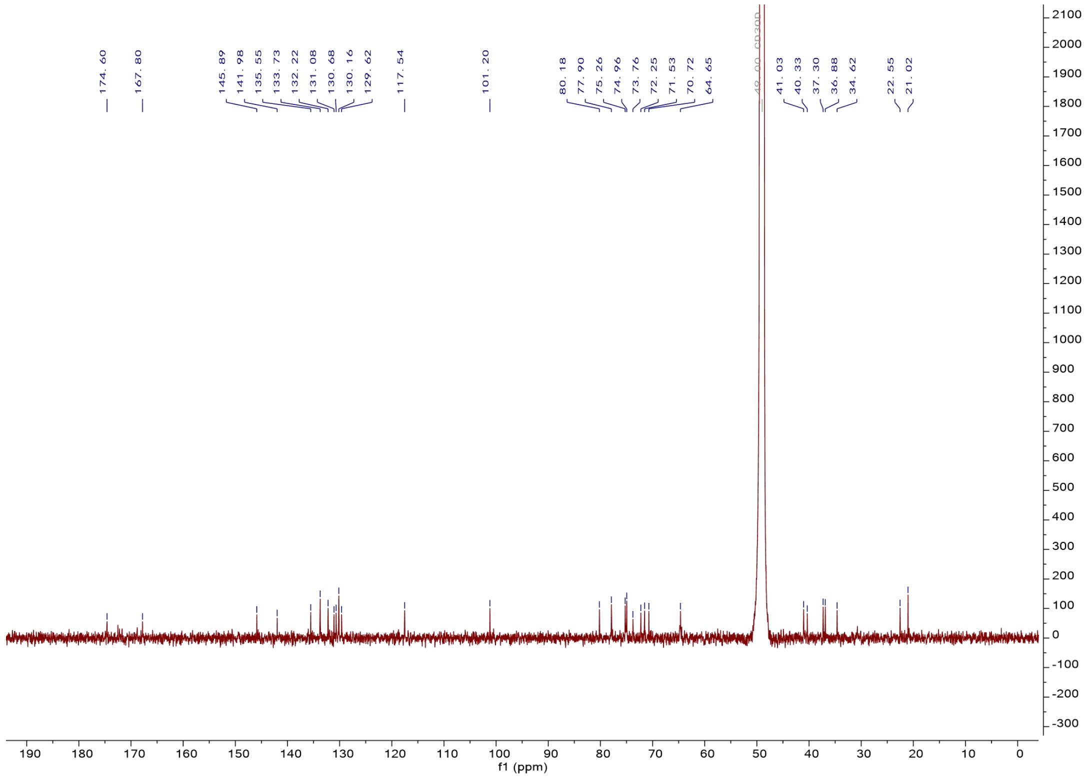

Figure S22. ^{13}C NMR spectrum of compound **3** (CD_3OD, 175MHz).

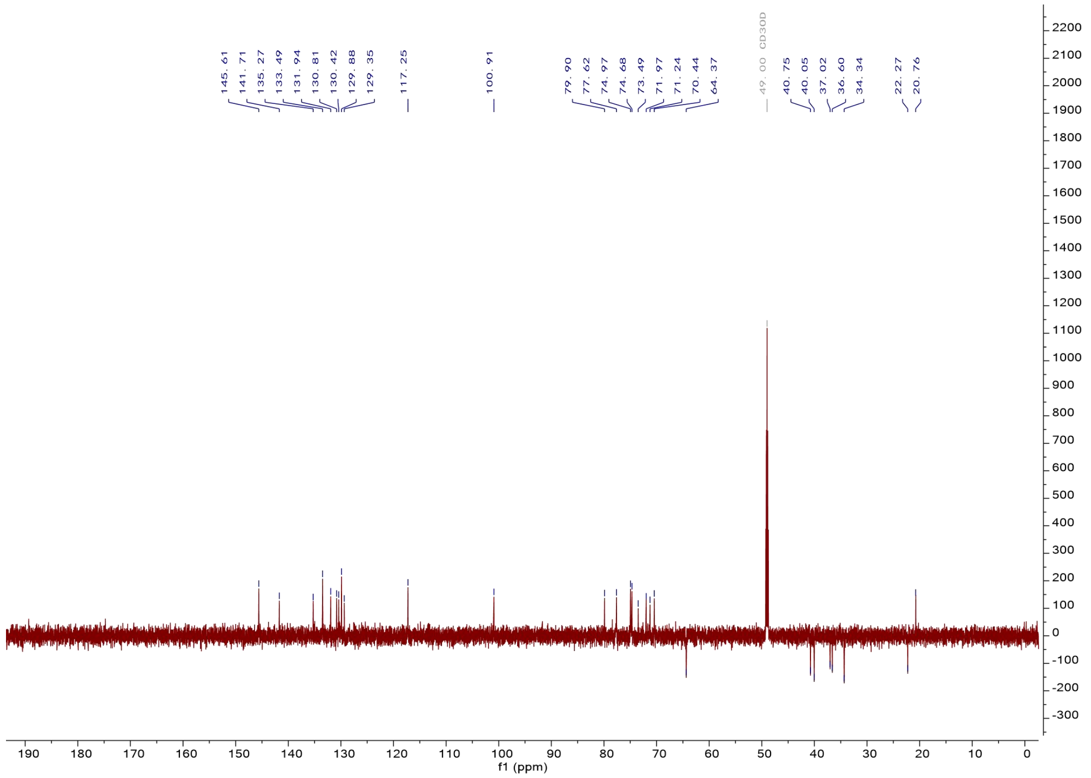

Figure S23. ^{13}C-DEPT135 NMR spectrum of compound **3** (CD_3OD, 175MHz).

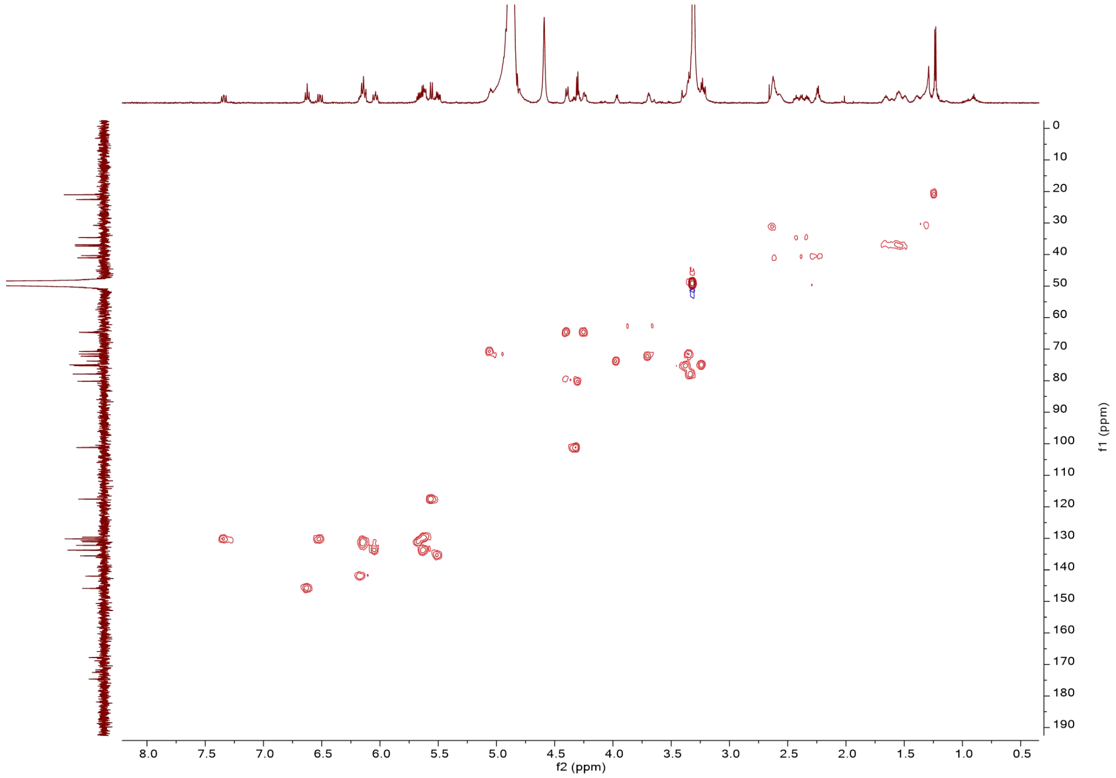

Figure S24. HMQC spectrum of compound **3** (CD_3OD).

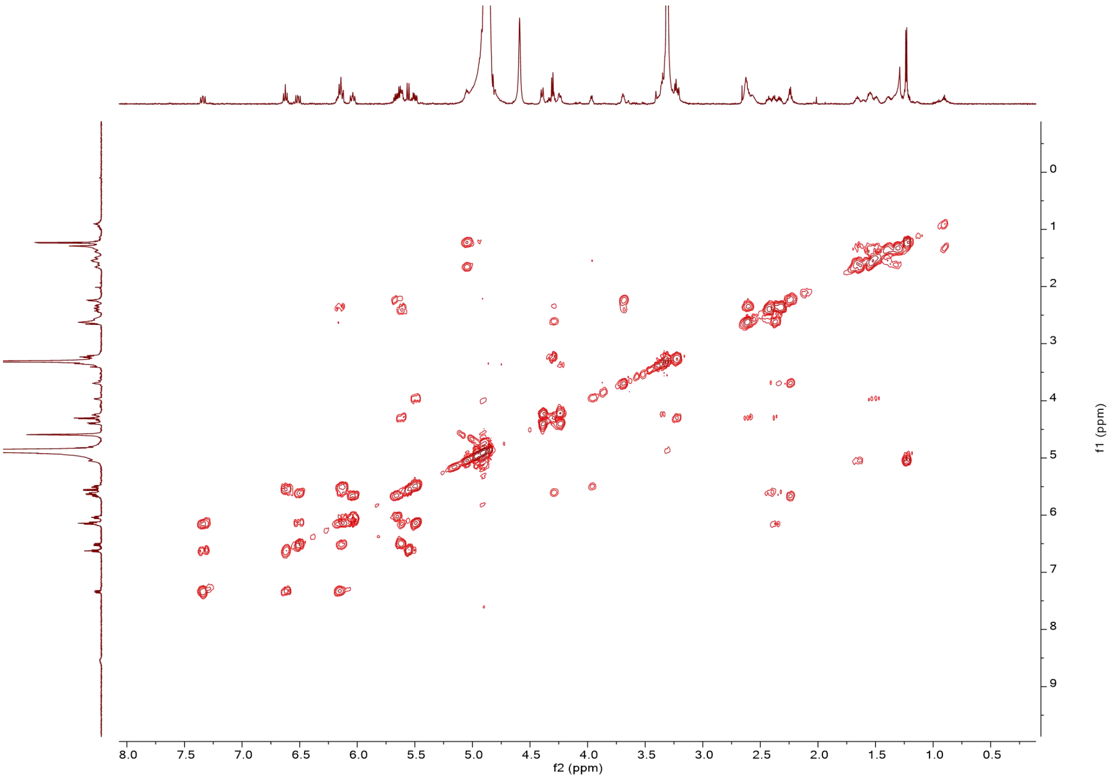

Figure S25. ^{1}H-^{1}H COSY spectrum of compound **3** (CD_3OD).

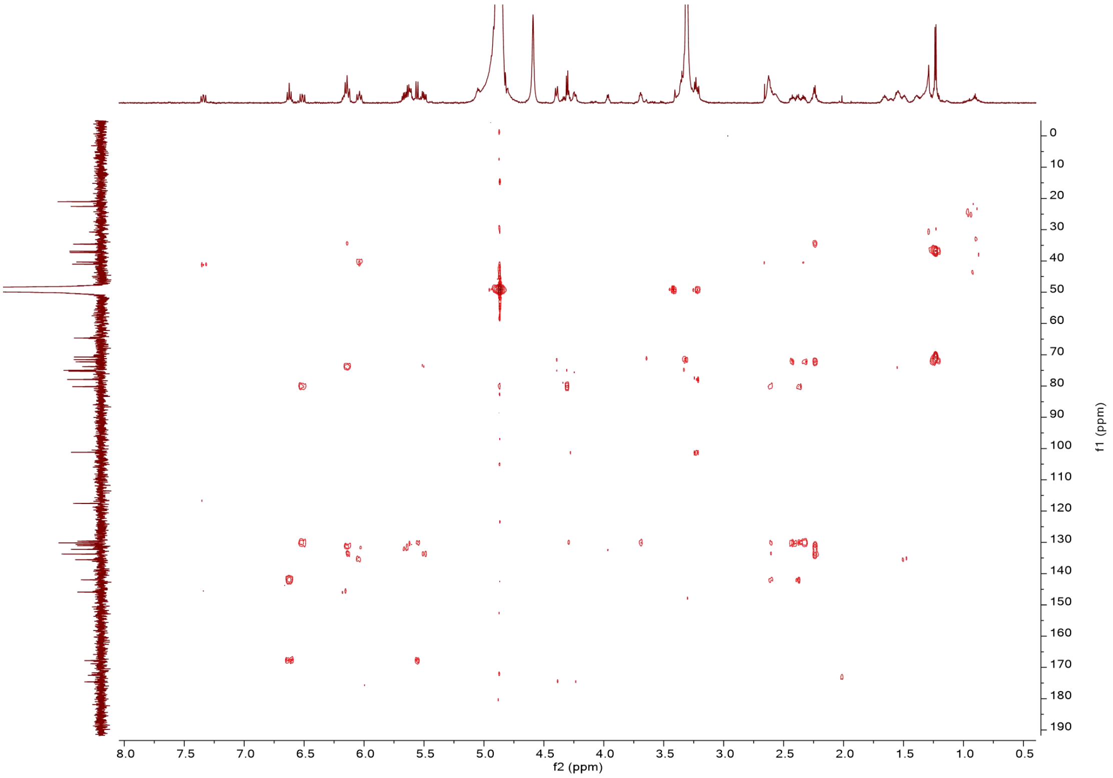

Figure S26. HMBC spectrum of compound **3** (CD_3OD).

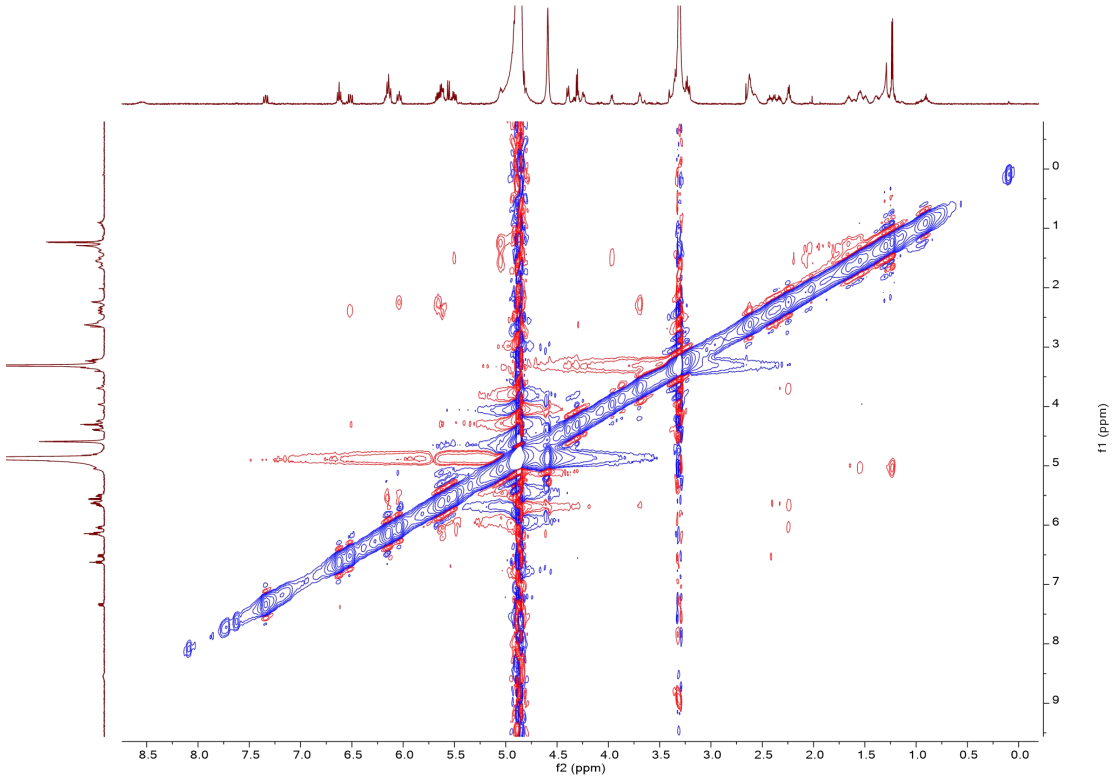

Figure S27. NOESY spectrum of compound **3** (CD_3OD).

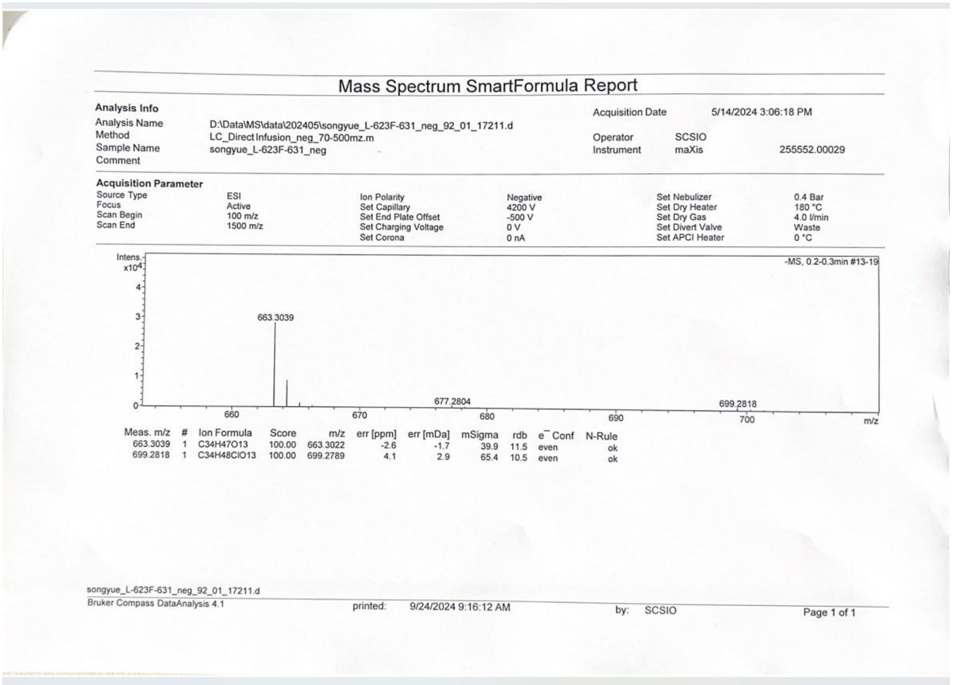

Mass Spectrum SmartFormula Report

Analysis Info

Analysis Name	D:\Data\MS\data\202405\songyue_L-623F-631_neg_92_01_17211.d	Acquisition Date	5/14/2024 3:06:18 PM	
Method	LC_Direct Infusion_neg_70-500mz.m	Operator	SCSIO	
Sample Name	songyue_L-623F-631_neg	Instrument	maXis	255552.00029
Comment				

Acquisition Parameter

Source Type	ESI	Ion Polarity	Negative	Set Nebulizer	0.4 Bar
Focus	Active	Set Capillary	4200 V	Set Dry Heater	180 °C
Scan Begin	100 m/z	Set End Plate Offset	-500 V	Set Dry Gas	4.0 l/min
Scan End	1500 m/z	Set Charging Voltage	0 V	Set Divert Valve	Waste
		Set Corona	0 nA	Set APCI Heater	0 °C

Meas. m/z	#	Ion Formula	Score	m/z	err [ppm]	err [mDa]	mSigma	rdb	e⁻ Conf	N-Rule
663.3039	1	C34H47O13	100.00	663.3022	-2.6	-1.7	39.9	11.5	even	ok
699.2818	1	C34H48ClO13	100.00	699.2789	4.1	2.9	65.4	10.5	even	ok

songyue_L-623F-631_neg_92_01_17211.d
Bruker Compass DataAnalysis 4.1 printed: 9/24/2024 9:16:12 AM by: SCSIO Page 1 of 1

Figure S28. HR-ESI-MS spectrum of compound **3**.

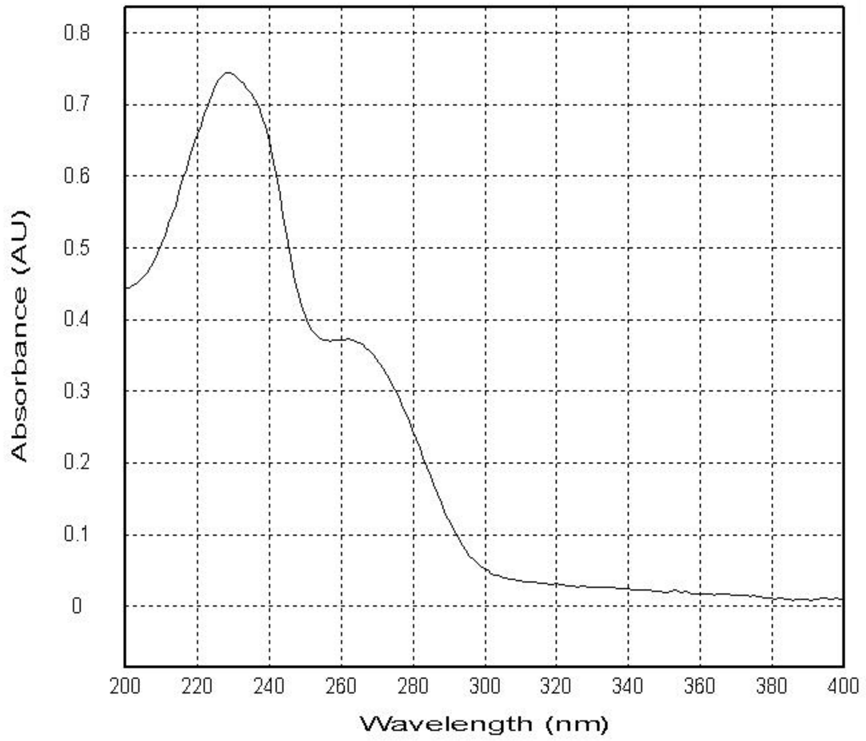

Figure S29. UV spectrum of compound **3**.

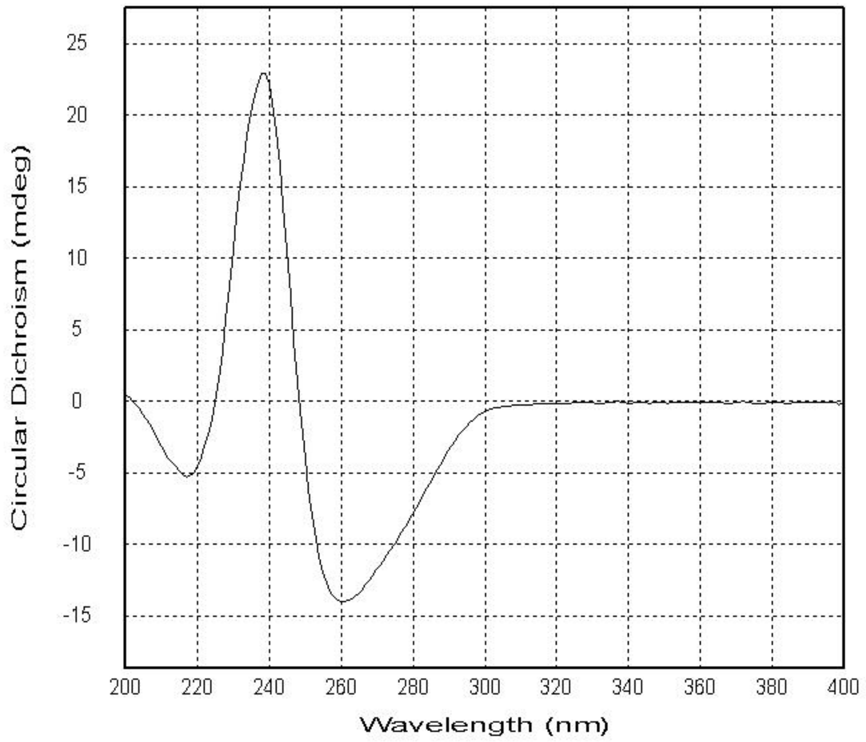

Figure S30. CD spectrum of compound **3**.

**Strains**	**relevant genotype and/or characteristics**
PAO1	ATCC *Pseudomonas aeruginosa*
PAO1-*pqsA-gfp*	PAO1 containing *pqsA-gfp* (ASV) translational reporter fusion
PAO1*ΔlasIΔrhlI*	PAO1 *lasI* and *rhlI* mutant

Table S1. The tested *P. aeruginosa* strains used in this study.

Physicochemical data of known compounds **4**–**5**.

Stellarine A **(4):** Green powder, ^{1}H NMR (700 MHz, DMSO-d_6) δ_H 12.20 (s, 1H, 9-NH), 9.14 (s, 1H, H-4), 8.44 (d, J = 7.6 Hz, 1H, H-5), 8.25 (s, 1H, 11-NH_2), 7.84 (d, J = 7.9 Hz, 1H, H-8), 7.63 (t, J = 7.4 Hz, 1H, H-7), 7.35 (t, J = 7.3 Hz, 1H, H-6), 2.85 (s, 3H, H-13). ^{13}C NMR (176 MHz, DMSO-d_6) δ_C 201.2(C-12), 166.7(C-10), 142.3(C-13), 134.99(C-1), 134.93(C-9a), 131.5(C-4a), 129.3(C-7), 122.2(C-5), 120.9(C-6, 4b), 113.4(C-8), 25.8(C-15).

9*H*-pyrido[3,4-*b*]indole-3-carboxylic acid **(5):** Yellow powder, ^{1}H NMR (700 MHz, DMSO-d_6) δ_H 11.64 (s, 1H, 9-NH), 8.82 (s, 1H, H-4), 8.39 (s, 1H, H-5), 7.83 (s, 1H, H-8), 7.63 (s, 1H, H-7), 7.44 (s, 1H, H-3′), 7.33 (s, 1H, H-6), 6.61 (s, 1H, H-4′), 4.68 (s, 2H, H-6′). ^{13}C NMR (176 MHz, DMSO-d_6) δ_C 167.4(C-10), 157.2(C-5′), 151.1(C-2′), 141.5(C-8a), 139.1(C-3), 132.1(C-1), 131.7(C-9a), 129.95(C-4a), 128.8(C-7), 121.98(C-5), 121.0(C-4b), 120.5(C-6), 115.5(C-4), 112.9(C-8), 110.98(C-3′), 109.2(C-4′), 55.94(C-6′).
